# Functional Metagenomics Unveils a Multifunctional Glycosyl Hydrolase from the Family 43 Catalysing the Breakdown of Plant Polymers in the Calf Rumen

**DOI:** 10.1371/journal.pone.0038134

**Published:** 2012-06-25

**Authors:** Manuel Ferrer, Azam Ghazi, Ana Beloqui, José María Vieites, Nieves López-Cortés, Julia Marín-Navarro, Taras Y. Nechitaylo, María-Eugenia Guazzaroni, Julio Polaina, Agnes Waliczek, Tatyana N. Chernikova, Oleg N. Reva, Olga V. Golyshina, Peter N. Golyshin

**Affiliations:** 1 CSIC, Institute of Catalysis, Madrid, Spain; 2 CSIC, Instituto de Agroquímica y Tecnología de Alimentos, Valencia, Spain; 3 HZI-Helmholtz Centre for Infection Research, Braunschweig, Germany; 4 Insect Symbiosis Research Group, Max Planck Institute for Chemical Ecology, Jena, Germany; 5 School of Biological Sciences, Bangor University, Gwynedd, United Kingdom; 6 Department of Biochemistry, University of Pretoria, Pretoria, South Africa; 7 Centre for Integrated Research in the Rural Environment, Aberystwyth University-Bangor University Partnership (CIRRE), Penglaid Campus, Aberystwyth, Ceredigion, United Kingdom; Missouri University of Science and Technology, United States of America

## Abstract

Microbial communities from cow rumen are known for their ability to degrade diverse plant polymers at high rates. In this work, we identified 15 hydrolases through an activity-centred metagenome analysis of a fibre-adherent microbial community from dairy cow rumen. Among them, 7 glycosyl hydrolases (GHs) and 1 feruloyl esterase were successfully cloned, expressed, purified and characterised. The most striking result was a protein of GH family 43 (GHF43), hereinafter designated as R_09-02, which had characteristics very distinct from the other proteins in this family with mono-functional β-xylosidase, α-xylanase, α-L-arabinase and α-L-arabinofuranosidase activities. R_09-02 is the first multifunctional enzyme to exhibit β-1,4 xylosidase, α-1,5 arabinofur(pyr)anosidase, β-1,4 lactase, α-1,6 raffinase, α-1,6 stachyase, β-galactosidase and α-1,4 glucosidase activities. The R_09-02 protein appears to originate from the chromosome of a member of *Clostridia,* a class of phylum *Firmicutes*, members of which are highly abundant in ruminal environment. The evolution of R_09-02 is suggested to be driven from the xylose- and arabinose-specific activities, typical for GHF43 members, toward a broader specificity to the glucose- and galactose-containing components of lignocellulose. The apparent capability of enzymes from the GHF43 family to utilise xylose-, arabinose-, glucose- and galactose-containing oligosaccharides has thus far been neglected by, or could not be predicted from, genome and metagenome sequencing data analyses. Taking into account the abundance of GHF43-encoding gene sequences in the rumen (up to 7% of all GH-genes) and the multifunctional phenotype herein described, our findings suggest that the ecological role of this GH family in the digestion of ligno-cellulosic matter should be significantly reconsidered.

## Introduction

Glycosyl hydrolases (GHs) are enzymes that are involved in the degradation of plant polymers and are produced by diverse prokaryotic and eukaryotic organisms. In the past decade, approximately 4679 and 49099 GHs homologues (with and without carbohydrate-binding domains, respectively) were described, and their sequences become available in public databases [1]. The microbes that populate the gastrointestinal (GI) tracts of herbivorous animals are continuously exposed to a strong diet-driven selective pressure by chemically diverse and complex plant polymeric compounds and constantly compete for the available sources of nutrition. As a consequence, these microbes display complex hydrolytic networks containing more putative GH homologues, as compared to those found in soil or water samples, with approximately 1.5 and 0.3% of the total genes, respectively [2]–[4].

The study of microbial (meta-) genomes arising from these specific environments contribute to our understanding of the hydrolytic enzyme networks operating in both the individual microbes and the entire GI microbial communities, and increases our chances to identify enzymes with new activities [5]–[16]. Accordingly, such studies are of a high ecological relevance [16]–[18]; it is noteworthy that previous investigations on ester-hydrolases and polyphenol oxidases from ruminal metagenomes [19], [20] have revealed enzymes representing novel functionalities.

In present study, we have identified 14 GH enzymes and 1 feruloyl esterase from a fibre-adherent microbial community from calf rumen using functional screens with sugar derivatives as the substrates. We performed an in-depth characterisation of 8 purified enzymes and discovered a multifunctional member of the GH family 43 (GHF43). These findings are discussed in the context of carbohydrate metabolism, the evolution of enzymes toward the ability to convert diverse and chemically complex compounds from plant-derived polymers and the ecological significance of such enzymes for the adaptation of microbial communities to thrive in this very peculiar environmental niche.

**Figure 1:**
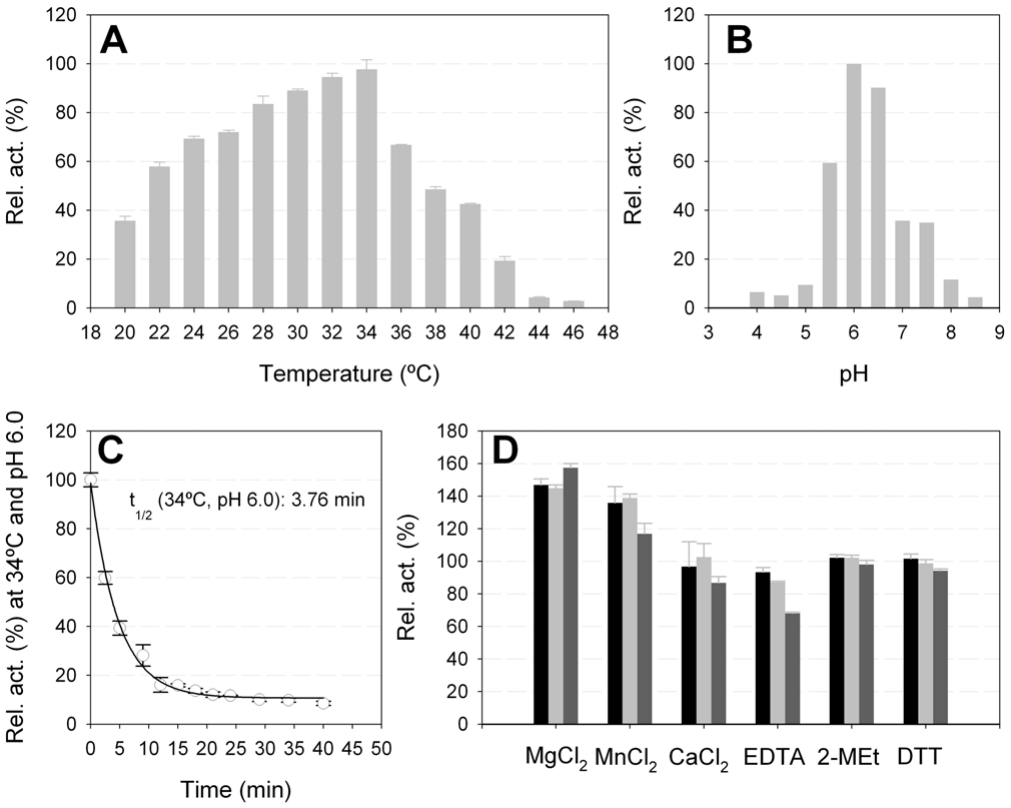
Temperature (A) and pH (B) optima and stability (C and D) of the purified GHF43 R_09-02 protein. The parameters were determined using *p*NPβX as the substrate. (**A**) For the optimum temperature determination, the pH was adjusted to 6.0 (sodium acetate 20 mM). (**B**) The optimum pH was determined in the range of pH 4.0–9.0 at 34°C. The buffers (100 mM) used were as follows: acetate (pH 4.0–6.0), MES (pH 6.0–7.0), HEPES (pH 7.0–8.0) and Tris-HCl (pH 8.0–9.0). In both cases, the *k*_cat_ value was determined using an [E] ranging from 0 to 12 nM and a substrate concentration of 70 mM. Activity at 100% refers to 230.3±13.7 s^−1^ at pH 6.0 and 34°C. (**C**) The time lost normalised quantification of the R_09-02 activity levels (with *p*NPβX) at 34°C and pH 6.0 (sodium acetate 20 mM) is shown. Protein (1.5 μg) was incubated, and the activity was determined as described in the **Methods**. (**D**) The effect of chemical reagents and metal ions on the hydrolase activity (*p*NPβX). The concentrations of the various chemicals ranged from 2 mM (black) and 5 mM (light grey) to 10 mM (dark grey), and the relative activities were defined using the activity ratio without the added chemicals. The optimal pH (6.0) and temperature (34°C) were used in the assays. All of the measurements were analysed in triplicate, and error bars are indicated. The error bars represent the standard deviation of three replicates from a single protein preparation.

**Table 1:** Kinetic parameters of the purified R_09-02 enzyme.

Substrate	*K*_m_ (mM)^1^	*k*_cat_ (s^−1^)^a^	*k*_cat_/*K*_m_ (s^−1^M^−1^)^a^
*p*NPαAf	0.57±0.15	5.38±0.26	9.4·10^3^
*p*NPαAp	6.98±0.79	230.3±13.7	3.3·10^4^
*p*NPβX	4.40±0.76	45.38±2.99	1.0·10^4^
1,4-β-Xylobiose	0.012±0.002	6.10±0.08	5.1·10^5^
1,4-β-Xylotriose	0.29±0.02	1.99±0.27	6.9·10^3^
1,4-β-Xylotetraose	0.33±0.07	0.67±0.17	2.0·10^3^
1,4-β-Xylopentaose	1.58±0.19	0.61±0.47	387
1,4-β-Xylohexaose	4.46±0.98	0.37±0.15	83
1,5-α-L-Arabinobiose	0.027±0.01	5.52±0.49	2.0·10^5^
1,5-α-L-Arabinotriose	0.10±0.05	0.59±0.08	5.9·10^3^
1,5-α-L-Arabinotetraose	4.23±0.74	0.11±0.04	26.00
*p*NPβGal	0.37±0.12	3.22±0.30	9.7·10^3^
*p*NPαG	4.84±1.27	0.25±0.02	52
*p*NPαMal	9.74±0.41	0.13±0.01	13
Maltose	2.57±0.19	0.06±0.01	23
Maltotriose	0.44±0.13	1.00±0.13	2.3·10^3^
Maltotetraose	0.53±0.17	0.54±0.03	1.0·10^3^
Maltopentaose	1.64±0.21	0.32±0.02	195
Maltohexaose	3.27±0.23	0.30±0.01	92
Maltoheptaose	6.20±0.18	0.06±0.01	9.7
Lactose	0.050±0.0039	4.24±0.16	8.5·10^4^
Raffinose	0.035±0.004	2.91±0.15	8.3·10^4^
Stachyose	1.43±0.84	1.70±0.02	1.1·10^3^

a *K*_m_, *k*_cat_ and *k*_cat_ /*K*_m_ values were obtained at pH 6.0 (sodium acetate 20 mM) and 34°C with [E]_o_ =  0–12 nM and a substrate concentration ranging from 0 to 150 mM.

**Figure 2:**
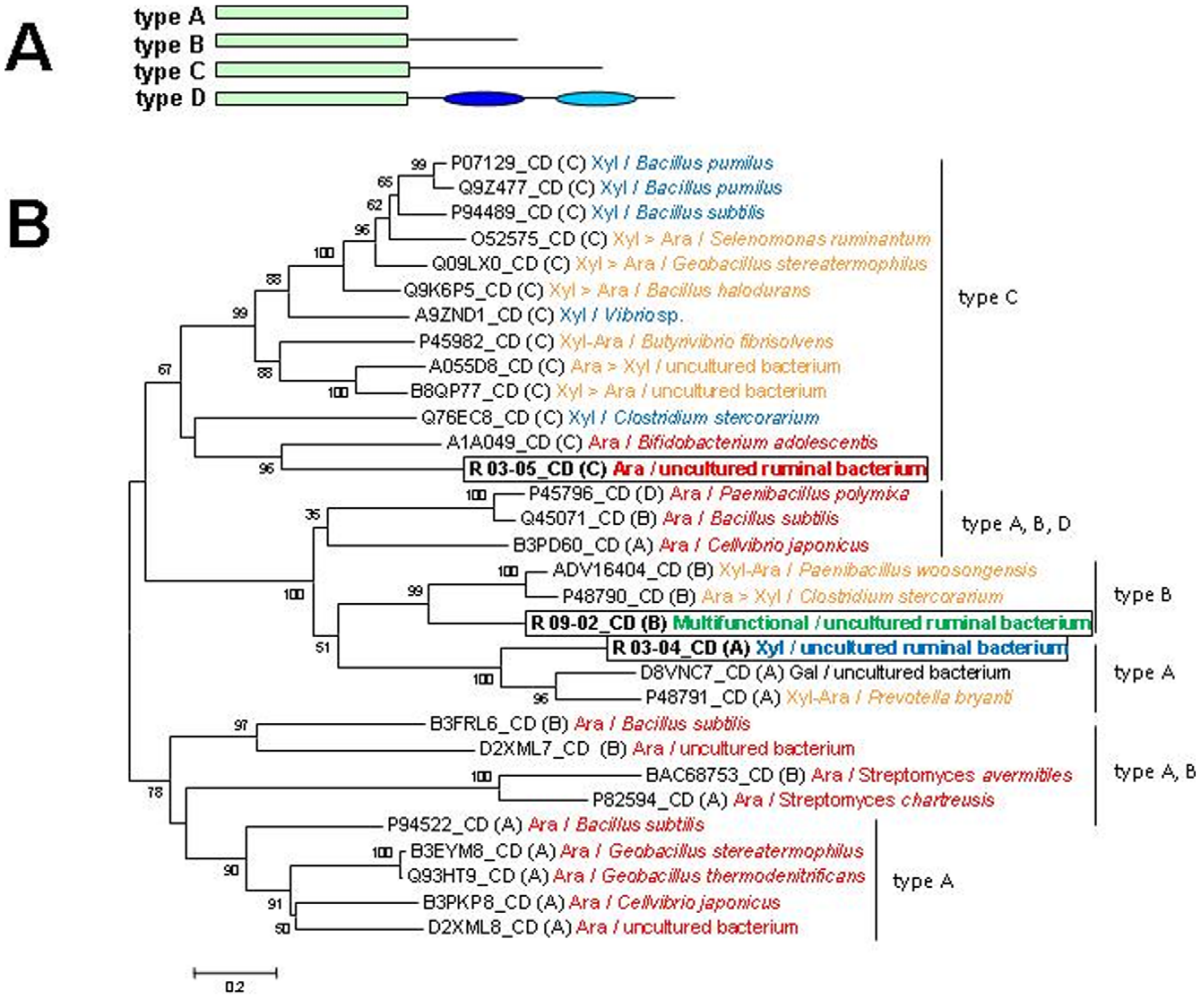
Phylogenetic and modular characteristics of the GHF43 proteins identified in the present study. (**A**) The scheme of modular arrangements in the biochemically characterised GHF43 enzymes. The catalytic module is represented with a green box. The single representative of type D (Uniprot code P45796) is predicted to contain domains in the C-terminal extension a CBM6 and a CBM36 module (dark and light blue ovals, respectively) [42]. In one case of the type B enzymes (Uniprot code Q45071), a CBM6 domain is predicted as a Pfam hit in the C-terminal domain. (**B**) Phylogenetic tree of the catalytic domains of the biochemically characterised GHF43 enzymes. The GHF43 catalytic modules were selected according to the predictions as Pfam hits, before Clustal alignment. The modular type (according to the scheme in [A]) and the Uniprot or NCBI (underlined) accession code of the original protein are indicated in each case. The GHF43 enzymes analysed in this study (R_03-04, R_03-05, R_09-02) are included and highlighted with a box. Those enzymes include xylosidases (Xyl), arabinosidases (Ara), bifunctional xylosidases/arabinosidases with similar activities for both substrate types (Xyl-Ara) or with certain preference for one or another (Xyl > Ara or Ara > Xyl), galactosidase (Gal) and the multifunctional R_09-02; enzymes with more than one catalytic domain were not included. The letters in brackets indicate the type of GH. The numbers on the branches indicate bootstrap values greater than 50%. Phylogenetic analysis of protein sequences was conducted with *MEGA* 4.0 software [43] using the Neighbor-Joining treeing method and Poisson correction. **Table S7** contains a list of bibliographic records that provided experimental support for enzymes described in the Figure.

**Figure 3:**
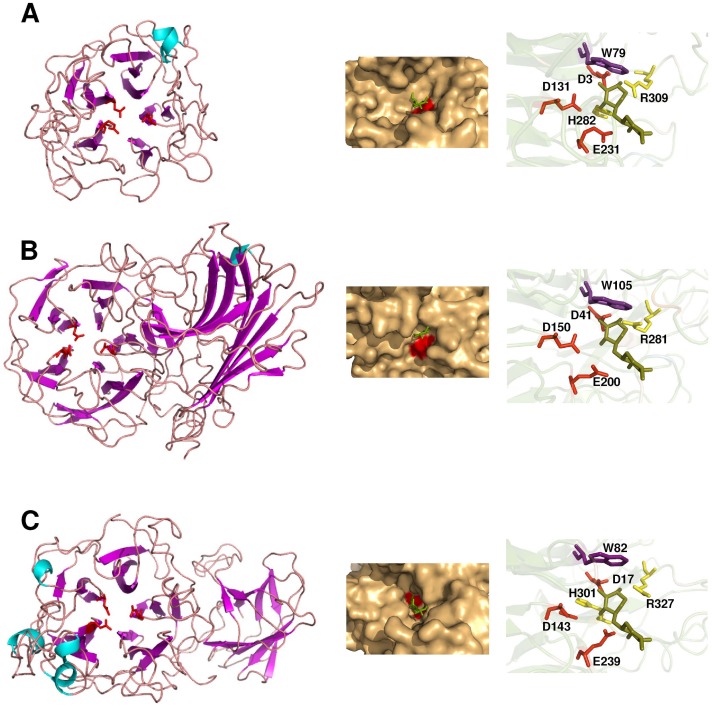
Structural models of R-03_04 (A), R-03_05 (B) and R-09_02 (C). The putative catalytic residues (general acid, base and transition state stabilisers) are depicted in red. The left panel shows the overall folding of the protein. The right panel is a close-up view of the catalytic site, indicating the catalytic residues and other highly conserved residues among the GHF43 enzymes that may establish polar (yellow) or hydrophobic (violet) contacts with the substrate at the −1 subsite. The middle panel shows the solvent-accessible surface close to the catalytic site. As a reference, the location of a xylobiose molecule (green) is given according to the structural superimposition with the β-xylosidase from *Geobacillus stearothermophilus* (PDB code 2EXJ).

**Figure 4:**
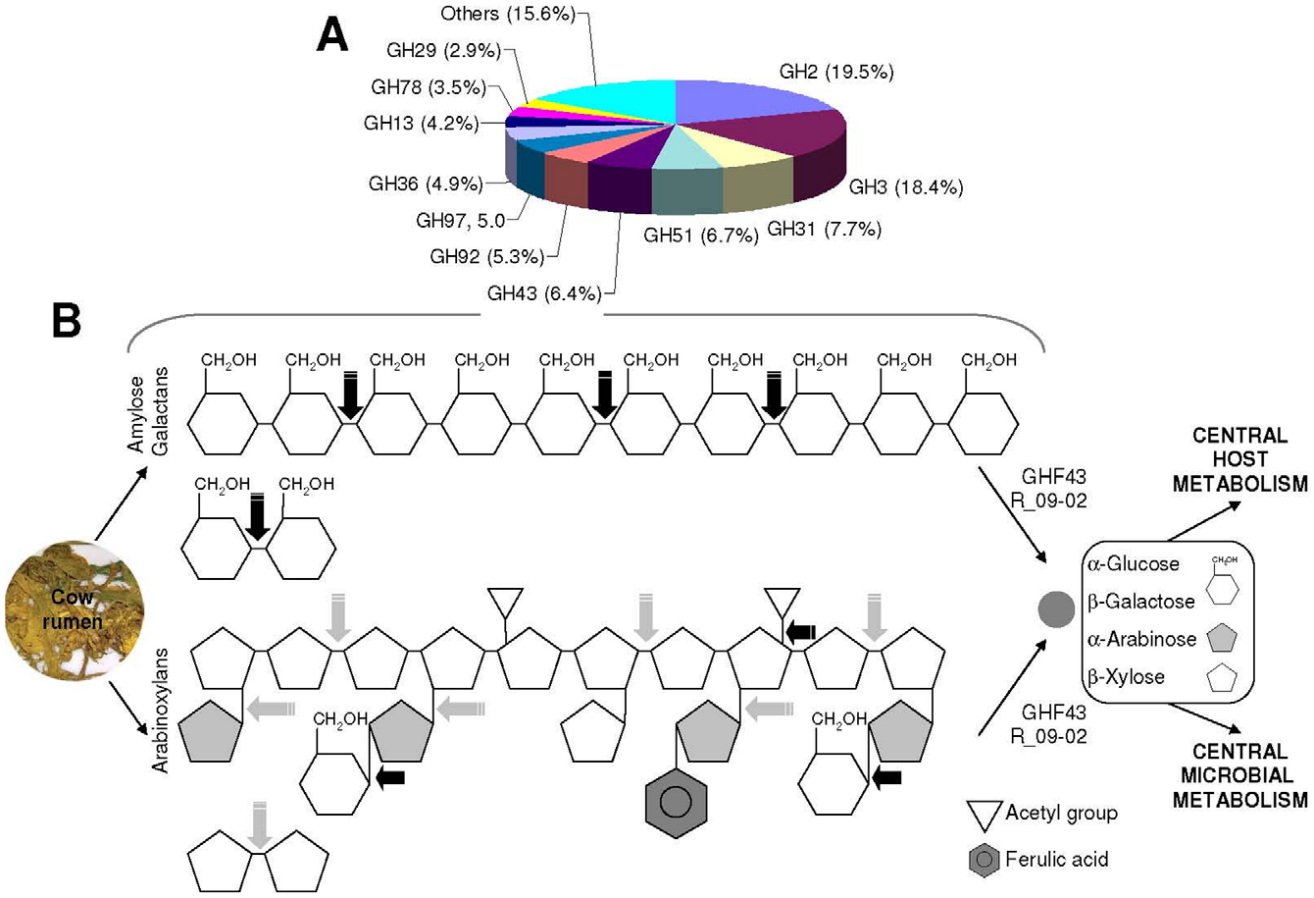
Contribution of GHF43 proteins to plant polymer hydrolysis in the rumen. (**A**) The relative abundance and distribution of glycosyl hydrolase families in the metagenome from the bovine rumen microbiome. The data include the pyrosequencing data of 4 metagenomic samples, including fibre-adherent and pooled liquid [10]. (**B**) New pathways of pentose and hexose digestion by the R_09-02-like enzymes in bovine rumen. The scheme indicates that R_09-02 may contribute to the digestion of arabinoxylans (a common activity associated with GHF43 enzymes: grey arrows) and gluco- and galactooligosaccharides (black arrows) derived from amylose/starch and galactans.

**Figure 5:**
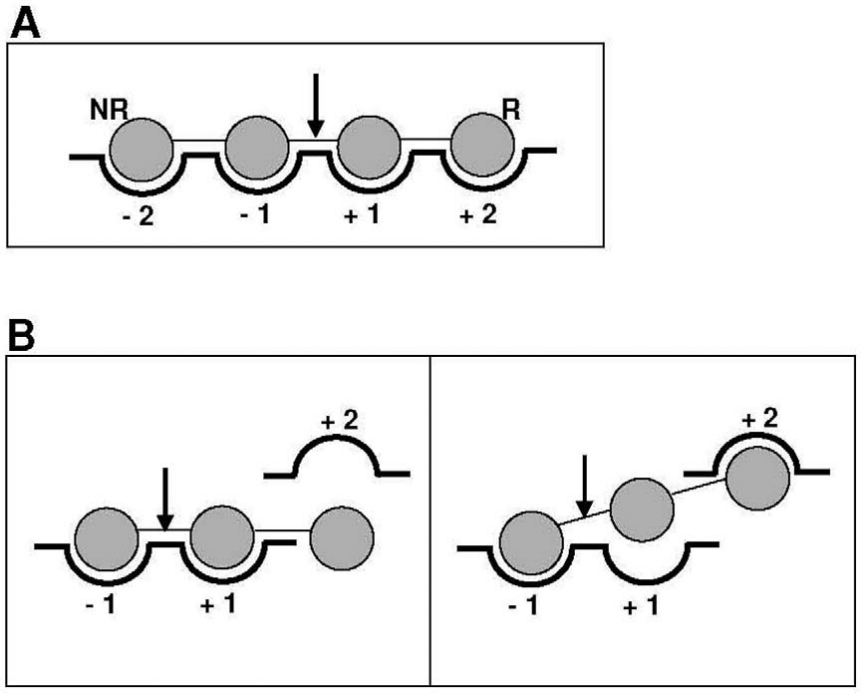
Sugar-binding sub-sites. (**A**) Schematic view of the nomenclature for the sugar-binding subsites in glycosyl hydrolases [41], with an oligosaccharide represented by the connected grey circles oriented from the non-reducing (NR) to the reducing (R) end. The arrow indicates the glycosidic bond that is susceptible to cleavage by the enzyme. (**B**) The proposed subsite distribution in R_09-02. Xylo-, arabino-, lacto- and malto-oligosacharides may share a promiscuous subsite −1. The first three may be oriented toward subsite +1, without a strong contribution of subsite +2 for their binding (left panel), whereas malto-oligosacharides may skip subsite +1 and be oriented toward subsite +2 (right panel).

## Methods

Total DNA was extracted from a fibre-adherent ruminal microbial community of one New Zealand dairy cow, as described in our previous study [19], using the G'NOME® DNA Isolation Kit (Qbiogene, Heidelberg, Germany). A brief description is presented below. For more details see the **Methods S1** and **References S1.**

### Metagenomic library construction and enzyme screening

Purified and size-fractioned DNA was ligated into the pCCFOS fosmid vector and further cloned in *Escherichia coli* EPI300-T1^R^ according to the instructions of Epicentre Biotechnologies (WI, USA) and a procedure described earlier [21]. Fosmid clones (12288) harbouring approximately 490 megabasepairs (Mbp) of community genomes were arrayed using the QPix2 colony picker (Genetix Co., UK) and grown in 384-microtitre plates containing Luria Bertani (LB) medium with chloramphenicol (12.5 μg/ml) and 15% (*v/v*) glycerol and stored at −80°C.

To screen for GH activity, the clones were plated onto large (22.5×22.5 cm) Petri plates with LB agar containing chloramphenicol (12.5 μg/ml) to create an array of 2304 clones per plate. Each library was screened for the ability to hydrolyse *p-*nitrophenyl (*p*NP) α-L-arabinofuranoside (*p*NPαAf), *p*NP-α-galactopyranoside (*p*NPαGal), *p*NP-α-L-rhamnopyranoside (*p*NPαR) and carboxymethyl cellulose (CMC). For screens based on *p*NP-like substrates, Induction Solution (Epicentre Biotechnologies; WI, USA) was added after an overnight incubation, as recommended by the supplier, to induce a high fosmid copy number. The CMC-active fosmids were screened on agar plates supplemented with 1% (w/v) substrate and Congo red water solution [19]. The positive clones were selected and fully or partially (after sub-cloning in the pUC19 vector) sequenced at the Göttingen Genomics Laboratory (Germany) or by primer walking from both ends at Secugen S.L. (Madrid).

### Cloning, expression, purification and characterisation of plant polymeric substance hydrolases

All genes for recombinant enzymes used in the present study were PCR-amplified using custom oligonucleotide primers and were cloned, expressed and purified as described in the **Methods S1.**

For the enzyme characterisation, the absorbance was measured using a BioTek Synergy HT spectrophotometer under the following conditions: [E]_o_ = 0–12 nM, [substrate] ranging from 0 to 50 mM in 100 mM buffer, *T* =  40°C. For the hydrolysis of the *p*NP derivatives, the corresponding volume of a *p*NP derivative stock solution (120 mM) in the appropriate buffer was incubated for 2–10 min (with the exception that 30 s were used for the assay for R_09-02) with 12 nM enzyme diluted in 200 µl of 100 mM buffer and measured at 405 nm in 96-well microtiter plates. The substrates tested included *p-*nitrophenyl (*p*NP) α-L-arabinofuranoside (*p*NPαAf), *p*NP-α- and *p*NP-β-galactopyranoside (*p*NPαGal and *p*NPβGal), *p*NP-α- and *p*NP-β-xylopyranoside (*p*NPαX and *p*NPβX), *p*NP-β-D-glucopyranoside (*p*NPβG), *p*NP-β-D-cellobioside (*p*NPβC), *p*NP-α-L-rhamnopyranoside (*p*NPαR) and *p*NP-α- and *p*NP-β-arabinopyranoside (*p*NPαAp and *p*NPβAp). For oligosaccharides other than the activated *p*NP derivatives, the level of released glucose was determined using a glucose oxidase kit (Sigma-Fluka-Aldrich Co., St. Louis, MO, USA). For xylo- and arabino-oligosaccharides, the levels of released xylose and arabinose were determined using the D-xylose and lactose/galactose (Rapid) assay kits from Megazyme (Bray, Ireland). The hydrolysis of cinnamates (methyl ferulate and coumarate) was routinely measured, and the kinetic parameters were determined as described previously [22]. The initial rates were fitted to the Michaelis–Menten kinetic equation using non-linear regression to determine the apparent *K*_m_ and *k*_cat_; kinetic parameter calculations were performed based on the molecular masses described in **Table S1**.

The standard GH assay contained [E]_o_ = 12 nM, *p*NP derivative or substrate at 10 mM and 100 mM 4-(2-hydroxyethyl)piperazine-1-ethanesulfonic acid (HEPES) in a total volume of 200 μl at the optimal pH and temperature for each enzyme.

The standard feruloyl esterase assay contained [E]_o_ = 12 nM, methyl ferulate at 1 mM in 100 mM HEPES in a total volume of 200 μl at pH 8.0 and *T* = 40°C.

The pH and temperature optima were determined in the range of pH 4.0–10.0 and 5–60°C. The following buffers (100 mM) were used: acetate (pH 4.0–6.0), MES (pH 6.0–7.0), HEPES (pH 7.0–8.0), Tris-HCl (pH 8.0–9.0) and glycine (pH 9.0–10.0). pH was always adjusted at 25°C.

All of the values were determined in triplicate and were corrected for the spontaneous hydrolysis of the substrate. The results shown are the averages of three independent assays ± the standard deviation.

### *In silico* analysis of proteins and 3-D modelling

The MetaGeneMark tool with refined heuristic models for metagenomes (http://exon.gatech.edu/GeneMark/metagenome/index.cgi; [23]) was used to predict genes in the cloned DNA fragments (DNA sequences of fosmid clones were deposited with GenBank/EMBL/DDBJ under accession numbers JQ303337-JQ303344). The deduced proteins were analysed using blastp and psi-blast [24] against the non-redundant database sourced from the nucleotide (nr/nt) collection, reference genomic sequences (refseq_genomic), whole genome shotgun reads (wgs) and environmental samples (env_nt). The translation products were further analysed for protein domains using the Pfam-A [25] and Cluster of Orthologous Groups of protein (COG) databases [26]. Multiple sequence alignments were generated using the ClustalW tool (http://www.ebi.ac.uk/clustalw/index.html) integrated into the BioEdit software [27]. Structural alignments of the proteins homologous to GH obtained in this study were generated by GenTHREADER [28] and used to retrieve a model from the Swiss-Model server [29]. The PDB entries used as templates are described in the **Text S1**.

## Results

### Library screening and general enzyme characteristics

In the present work, the GHs were named according to the origin (rumen, R), fosmid ID and the number of the corresponding coding sequence (CDS) in the genomic fragment sequenced. The R library (12,288 fosmid clones) was screened for the ability to hydrolyse *p*NPαAf, *p*NPαGal, *p*NPαR and CMC. We identified eight positives (designated as r_01 to r_03 and r_05 to r_09). The fosmids with inserts r_01 (*p*NPαGal positive), r_02 (*p*NPαAf pos.) and r_03 (*p*NPαAf pos.) were fully sequenced, whereas those of r_05, r_06 (CMC pos.), r_07, r_08 (*p*NPαR pos.) and r_09 (*p*NPαAf pos.) were first subjected to shotgun sub-cloning. After subsequent activity screening with appropriate substrates, the inserts of positive sub-clones were then fully sequenced (**Table S1**).

Genes in the fully sequenced fosmid or plasmid clones were predicted using the MetaGeneMark tool [23], and the corresponding gene products were further subjected to a nr psi-blast analysis, which identified 14 GH- and 1 feruloyl esterase-like polypeptides (**Figure S1**; **Tables S2, S3, S4**) and 13 additional accessory enzymes acting on carbohydrates (**Table S5**). We observed a high sequence similarity between the GHs and other putative genes from clones r_01 and r_06 to r_09 and the proteins from organisms of the phylum *Firmicutes*, although the average GC content in those clones was approximately 60% (**Table S2**); other clones (r_02, r_03 and r_05) possessed genes whose products were related to the proteins from representatives of the phylum *Bacteroidetes* (**Table S2**). Many representatives of the above phyla are culturable microorganisms found in the rumen and other regions of the GI tract and are thought to play key roles in the breakdown of proteins and carbohydrate polymers [10].

From these clones (except for r_05 and r_06, whose gene products could not be expressed in an active form), 7 putative GHs plus an additional esterase were cloned, expressed in *E. coli* and purified. Furthermore, their activities were tested with a battery of substrates (**Tables** **1** and **S6**) under optimal temperatures and pH values (**Figure 1**, **Figures S2** and **S3**) to determine the substrate(s) that were the most highly degraded. The presence or absence of putative secretion signal peptides, domain organisation (**Figure S4**) and 3-D models (**Figure S5**) were also analysed based on the sequence data. Seven of twelve *p*NP derivatives tested were hydrolysed by rumen community-derived enzymes (**Tables** **1** and **S6**), and the sequence analysis of the enzymes showed a similarity with specific protein domains of known GHs and esterases that are multimodular with diverse 3-D structures and substrate specificities (for details, see the **Text S1** and **References S1**). As expected for the screening substrates that were used, the major phenotypes identified were α- and β-galactosidase, α-arabinofuranosidase, α-rhamnosidase, β-xylosidase, β-cellobiase and β-glucosidase. The enzymes were characterised by a wide range of pH values ranging from 5.0 to 9.0, and seven of the enzymes exhibited their highest activity at approximately 50°C and showed a rapid loss of activity above this temperature. The only exception to this was with the R_09-02 enzyme, which was active at temperatures below 35°C. A complete description of the enzyme characteristics is provided in the **Text S1**.

Among all of the polysaccharide-degrading enzymes investigated, R_09-02 appeared to show atypical characteristics, and the extensive analysis of this enzyme is provided below.

### R_09-02 is a GHF43 enzyme with atypical activities

The insert of the r_09 DNA fragment (3264 bp; G+C content of 60.69%) contained three GHs, namely R_09-01, a putative truncated GHF43 protein, a xylosidase/arabinosidase (R_09-02) from GHF43 (most similar to those from *Bacteroides capillosus* and *Clostridium hathewayi*) and another truncated GHF1 β-galactosidase (R_09-03) (**Figure S1** and **Table S2H**).

R_09-02 has a deduced molecular mass of 54 939 Da and an estimated p*I* of 4.96. GHF43 comprises a large number of GHs from different organisms (1590 entries in GenBank, 482 in Uniprot and 33 in the PDB) that are known to act mainly on β-1,4(3)-xylans or α-1,3(5)-arabinans, with a few reported cases of galactosidases/galactanases. The analysis of the pure R_09-02 enzyme (a tetramer of approximately 200 kDa) using activated *p*NP derivatives revealed β-xylosidase, α-arabinofur(pyr)anosidase, β-galactosidase and, to a lesser extent, α-glucosidase activities (**Table** **1**), a profile that does not resemble the typical activity profile of enzymes from the GHF43 family (**Table S7**) [30]. In the enzymatic assay, the Michaelis-Menten constant (*K*_m_), the catalytic rate constant (*k*_cat_) and the catalytic efficiency (*k*_cat_/*K*_m_) values were determined (**Table** **1**). In terms of its catalytic efficiency, R_09-02 best hydrolysed *p*NP-α-arabinopyranoside (*p*NPαAp), followed by *p*NP-β-xylopyranoside (*p*NPβX), *p*NPαAf and *p*NP-β-galactopyranoside (*p*NPβGal): 71-, 43- and 5-fold greater *k*_cat_ values for *p*NPαAp, compared to *p*NPβGal, *p*NPαAf and *p*NPβX, respectively. A weak activity with *p*NPα-glucopyranoside (*p*NPαG) and *p*NPα-maltoside (*p*NPαMal) was detected, and a reduction in the catalytic efficiency with these substrates was mainly due to a 1771-fold reduction in the *k*_cat_ in comparison to *p*NPαAp.

The activity of the purified R_09-02 protein was further analysed against various oligosaccharides, as described in the **Methods** section (**Table** **1**): 1,4-β-xylo-oligosaccharides (degree of polymerisation [DP] from 2 to 7), 1,5-α-arabino-oligosaccharides (DP from 2 to 7), maltooligosaccharides (DP from 2 to 7), xyloglucan oligosacharides (DP ∼14), larch arabinogalactan, amyloid xyloglucan, and starch. The enzyme hydrolysed short 1,4-β-D-xylo-oligosaccharides with less than seven units and 1,5-α-L-arabino-oligosaccharides with less than five units. All of the substrates were completely degraded to the monosaccharides (not shown), suggesting an exo-mode of action for this glycosyl hydrolase. A [(*k*_cat_/*K*_m_)]_xylobiose_/[(*k*_cat_/*K*_m_)]_xylotriose_ factor of ∼74/1 was observed due to a 3-fold higher *k*_cat_ value coupled with a significantly (25-fold) lower *K*_m_ value for the shorter substrate. Similarly, a [(*k*_cat_/*K*_m_)]_arabinobiose_/[(*k*_cat_/*K*_m_)]_arabinotriose_ factor of ∼35/1 was observed, as the *K*_m_ and *k*_cat_ values for the disaccharide were 4-fold lower and 9-fold higher, respectively, when compared to the trisaccharide. As shown in **Table** **1** **,** xylobiose and arabinobiose were hydrolysed with similar *k*_cat_ values, although the former was somewhat preferred at lower substrate concentrations (∼2-fold lower *K*_m_), resulting in a 2-fold catalytic efficiency value. According to these data, the enzyme would be essentially bifunctional for xylobiose and arabinobiose at concentrations over 0.3 mM (more than 10-fold the *K*_m_ values). The lower activity with the longer substrates indicates the enzyme preference for shorter xylose- and arabinose-containing molecules.

1,4-α-Linked saccharides, ranging from maltose to maltoheptaose, were also used as substrates, albeit with lower efficiencies (less than 250-fold) when compared to those containing 1,4-β-xylose and 1,5-α-L-arabinose (**Table** **1**). The *k*_cat_/*K*_m_ value was the highest for maltotriose, followed by maltotetraose and, to a lesser extent, maltopentaose and maltohexaose, whereas maltose and maltoheptaose were poor substrates. No release of hydrolysis products was observed with substrates longer than maltoheptaose (including soluble starch). This substrate length specificity differs from that for xylose and arabinose-containing molecules for which the disaccharides were the preferred substrates.

We further demonstrated that R_09-02 hydrolysed the α-1,4 glucosidic bond of the disaccharide, lactose, and the α-1,6 bond in the trisaccharide, raffinose, which is the most preferred substrate after 1,4-β-xylobiose (six-fold rel. *k*_cat_/*K*_m_) and 1,5-α-arabinobiose (two-fold rel. *k*_cat_/*K*_m_). The tetrasaccharide, stachyose, was also hydrolysed, but R_09-02 was 74-fold less efficient with this substrate in comparison to raffinose, which was mainly due to a 41-fold increase in the *K*_m_, coupled with an approximately two-fold reduction in the *k*_cat_.

None of the other tested substrates was hydrolysed, suggesting that the natural substrates of R_09-02 are short oligosaccharides containing α-1,5 glucosidic bonds between two arabinoses, containing β-1,4 bonds between two xyloses, containing β-1,4 bonds between one galactose and one glucose, containing α-1,6 bonds between one galactose and one glucose and containing α-1,4 bonds between two glucoses. Altogether, the data confirmed the highly promiscuous behaviour of the R_09-02 protein. To the best of our knowledge, no GH with a similar biochemical profile has been described to date (**Table S7**) [30]. Therefore, the R_09-02 enzyme should be classified as a multifunctional GHF43 protein with β-xylosidase, α-arabinofur(pyr)anosidase, lactase, raffinase, stachyase, β-galactosidase and α-glucosidase activities.

The optimum activity for R_09-02 was observed within a narrow range of temperatures, with a relative activity higher than 80% of the maximum recorded occurring between 30 and 34°C, and within a narrow pH range (5.0–6.0) (**Figure 1** **,** panels A and B). This thermal sensitivity of the R_09-02 protein may explain why the protein was found mainly in inclusion bodies at 37°C and that high levels of the active protein could only be obtained when the expression was performed at temperatures lower than 28°C (**Figure S6**). The half-life of the enzyme at the optimal temperature of 34°C and optimal pH of 6.0 showed that the enzyme was quite unstable: the t_1/2_ was approximately 3.8 min (**Figure 1C**). For this reason, short incubation times (less than 1 min) were used to determine the kinetic parameters. The activity of R_09-02 was not affected by reducing agents, such as dithiothreitol and 2-mercaptoethanol (**Figure 1D**), suggesting that this enzyme (with 10 cysteine residues per monomer) does not contain any structurally relevant disulphide bonds. The addition of Mg^2+^ and Mn^2+^, but not Ca^2+^, increased the activity of the enzyme by approximately 1.5-fold. As structural calcium ions have been found in the β-sandwich module of other GHF43 enzymes [31], [32] or as a part of their catalytic sites [33], [34], the possibility that Mg^2+^ and Mn^2+^ may have similar structural roles cannot be ruled out. In fact, the original purified enzyme may contain such trace elements because the presence of the chelating agent, EDTA, at 10 mM inhibited the enzyme activity by approximately 67% (**Figure 1D**).

### 3D structural analysis of biochemically characterised GHF43

Most of the GHF43 enzymes analysed to date are either highly specific xylosidases or arabinofuranosidases, with a few cases of bifunctional xylosidases-arabinofuranosidases [35] and one reported galactosidase (see the CAZy database; [30]). The broad spectrum of activities found for R_09-02 led us to perform a phylogenetic comparison of the biochemically characterised GHF43 enzymes to determine the evolutionary relatedness of R_09-02 with counterparts that have different substrate specificities. Different modular arrangements were found that contained either a single catalytic module of approximately 300 amino acids (AA) or an N-terminal catalytic domain and an additional 150 AA, 230 AA or 280 AA–long C-terminal domain. These different modular topologies will be referred here as types A, B, C and D, respectively for simplification (**Figure 2**). Enzymes that contained more than one catalytic domain were excluded from this analysis. The amino acid sequence alignment was performed using only the corresponding GHF43 catalytic domains, based on the hits predicted by the Pfam database (http://pfam.sanger.ac.uk/). The catalytic domains of the type C enzymes were grouped together by the phylogenetic analysis, whereas types A, B and D apparently evolved independently (**Figure 2**). Because the phylogenetic clustering relies on modular properties rather than on the taxonomic placement of the organism, the separation of the above types was probably an ancient evolutionary event. According to this classification, R_09-02, R_03-04 and R_03-05 (GHF43 enzymes also identified herein; for details see the **text S1**) would belong to types B, A and C, respectively. The structural models of R_03-04, R_03-05 and R_09-02 (based on templates with PDB codes 3QED, 2EXI and 3C7G and sequence identities of 23.5%, 18.2% and 19.7%, respectively) revealed that R_03-04 contains a single catalytic module, whereas R_03-05 and R_09-02 contain a C-terminal β-sandwich domain (**Figure 3**, left panel). This accessory domain would be larger in R_03-05 enzyme, with a loop protruding into the active site. Indeed, the substrate-binding site of the structurally resolved type C enzymes includes residues from this β-sandwich [31], [36], and this may explain why the catalytic domain of these enzymes evolved independently. Most of the type C enzymes are known as xylosidases, whereas types A and B were identified mainly as arabinofuranosidases (**Table S7**) [30]. However, hydrolases from type C group exhibit also arabinofuranosidase activities, and the A and B types contain some xylosidases, indicating that the conversion of a xylosidase into an arabinofuranosidase and vice-versa is possible in any of these groups (**Table S7**) [30]. A set of residues that could potentially form hydrophobic or polar contacts with the substrate (W82, H301 and R327 in R-09_02) (**Figure 3**, right panel) is highly conserved within the GHF43 enzymes; the Arg residue is invariantly found in all of the characterised GHF43 enzymes, whereas, in some cases, the His is absent or the Trp is substituted with other hydrophobic residues (not shown), regardless of the main activity of the enzyme. Additionally, other hydrophobic residues (with a highly heterogeneous distribution among the GHF43 sequences) are found in the catalytic pocket and may contribute to the substrate binding. Either these additional residues or changes in the orientation of the lateral chain of conserved residues may be responsible for the differences in the substrate specificity. Whatever the case, R_09-02 belongs to a phylogenetic cluster that shows a quite divergent biochemical profile. This makes the identification of the motifs responsible for the R_09-02 promiscuity difficult, as it probably results from a combination of multiple sequence divergences. When more biochemical and structural data become available, this issue may be re-addressed.

## Discussion

In the present work, a functional metagenome library analysis was used to identify the components of the enzymatic machinery of the plant polymer-degrading microorganisms populating the rumen of a dairy cow. We detected 15 hydrolases and cloned, expressed, purified and characterised 8 of them (7 highly active GHs and 1 feruloyl esterase); these enzymes likely originated from the genomes of bacteria of the *Bacteroidetes* (e.g. **Figure S7**) and *Clostridia* classes that are known to be abundant in the ruminal environment.

The most intriguing finding was the discovery of a promiscuous GHF43 protein, named R_09-02. This enzyme was predicted to contain the typical β-propeller catalytic domain of GHF43 and a β-sandwich carbohydrate-binding domain that is structurally related to family 6 (CBM6). However, as a multifunctional α-1,5-arabinofur(pyr)anosidase, β-1,4-xylosidase, β-1,4 lactase, α-1,6 raffinase, α-1,6 stachyase, β-galactosidase and α-1,4 α-glucosidase, R_09-02 showed a unique substrate-specific pattern among the GHF43 enzymes characterised thus far [37]. The R_09-02 enzyme was highly active with both short α-arabinose- and β-xylose-containing substrates that are likely produced from the hemicellulose components of plant cell walls due to the action of xylanases. The enzyme was also active with short substrates that contained galactose and glucose units joined by β-1,4 and α-1,6 bonds, and to a lesser extent, with short α-1,4 maltooligosaccharides. R_09-02 demonstrated an absolute requirement of temperatures <35°C and notably retained only approximately 40% of its activity *in vitro* at the temperature common for the rumen milieu (38–40°C). Such a low temperature optimum is rather atypical for members of the GHF43 family [37], which optimally function at higher temperatures (50–60°C); this is consistent with the significant structural differences between R_09-02 and the other GHF43 enzymes. In respect to the substrate specificity and enzymatic activity, it is important to note that R_09-02 preferentially cleaved substrates with α-L-arabinose in the pyranose conformation. Taking into account that terminal arabinopyranose residues protect the cell walls from degradation by microbial α-L-arabinofuranosidase at the non-reducing terminus, the presence of R_09-02, which acts on substrates containing α-L-arabinose residues in the pyranose conformation, may enhance the efficiency of bacterial plant biomass degradation in the ruminal environment. From a biological point of view, the addition of R_09-02 to the set of “typical” GHF43 proteins may enhance the degradation of arabinan-containing polysaccharide mixtures (**Figure 4**). Furthermore, its wide substrate specificity suggests that R-09-02 (and related proteins) may also catalyse the hydrolysis of the mixed galactoside-glucoside components of plant seeds (e.g., galactans present in alfalfa; [34], [38] that are used in animal feed (**Figure 4**). Therefore, the presence and expression of R_09-02 and enzymes acting in a similar fashion seem to be beneficial for both the host and bacteria, even though the enzyme functions under sub-optimal temperature conditions. This issue is of a special ecological interest because we know that the genomes of many animals, such as the giant panda [39], lack the genes for enzymes that are needed to digest plant polymers. Furthermore, the energy uptake from plant biomass (e.g., [hemi-] cellulose substrates) is highly dependent on the metabolic capacity of the microbial community of the animals' GI tracts. Accordingly, the presence of enzymes acting on highly diverse substrates may be a beneficial factor for expanding the opportunities for niche colonisation of a certain bacterial group in the rumen or GI tract. At the same time, the presence of these enzymes could enhance the energetic value of the feed for the host. In this context, it should be noted that GHF43 proteins are among the most abundant families of GHs in (meta-) genome databases and encompass approximately 7% of all GHs identified in the bovine rumen (**Figure 4**) and 3% in the GI tracts of termites [10], [40].

For GHF43 in particular, and for GHs in general, the characteristics of the R_09-02 protein may also have implications from an evolutionary point of view. For these enzymes, substrate binding relies on specific subsites that interact with the oligo-(poly-)saccharide in the correct orientation for cleavage by the catalytic residues. According to the nomenclature established by Davies et al. [41], these subsites are designated with integer numbers from –n to +n (binding to the monomer units from the non-reducing to the reducing end, respectively), with the cleavage occurring between subsites −1 and +1 (**Figure 5A**). Thus, one of the most intriguing questions is how a gene encoding a GHF43 enzyme has evolved to have such broad substrate specificity. To answer this question, we performed a comparative analysis of the chemical structures of the different substrates (**Figure S8**) and the kinetic parameters for each of them (**Table** **1**). The relative *K*_m_ values for *p*NPαAp, *p*NPβX and *p*NPαG are very similar and much higher than those for *p*NPαAf and *p*NPβGal, suggesting that subsite −1 of R_09-02 has evolved to accommodate arabinofuranoside and galactopyranoside moieties with higher affinities. The divergence in the *k*_cat_, showing a clear preference toward hydrolysing the arabinose group in the pyranose conformation, may have resulted from different orientations of the glycosidic bond relative to the catalytic residues (nucleophile and acid/base catalyst) after the occupation of subsite −1. A comparison of the *K*_m_ values obtained with the *p*NP derivatives of the monosaccharides with those for the corresponding disaccharides may be used as an estimation of the affinities of subsite +1 for the different glycosyl groups. Thus *K*_m_ (*p*NPβX) / *K*_m_ (xylobiose) is 367, *K*_m_ (*p*NPαAf)/ *K*_m_ (arabinobiose) is 21, and *K*_m_ (*p*NPGal)/ *K*_m_ (lactose) is 7.4, whereas *K*_m_ (*p*NPαG)/ *K*_m_ (maltose) is only 1.9. This suggests that subsite +1 significantly contributes to the stable binding of the xylopyranoside, arabinofuranoside and glucopyranoside moieties from xylobiose, arabinobiose and lactose, respectively, but does not interact as tightly with the glucopyranoside group from maltose. Moreover, the nearly 6-fold decrease in the *K*_m_ value when comparing maltose with maltotriose may be indicative of subsite +2 efficiently coordinating the glucopyranoside moiety from malto-oligosacharides with more than 2 units. Based on the progression of *K*_m_ values, this subsite does not seem to contribute to the stable binding of xylo- and arabino-oligosacharides. From this evidence, we hypothesise that two alternative substrate-binding sites may coexist in R_09-02. Xylo-, arabino-, lacto- and malto-oligosacharides would share a promiscuous subsite −1; however, whereas the first three would be oriented toward a common subsite +1, the malto-oligosacharides may skip this site and be directed toward subsite +2 (**Figure** **5B**). Because the *K*_m_ of *p*NPαG and *p*NPαMal are similar, it may also be concluded that a subsite −2 is absent for the glucopyranoside moieties. A possible evolutionary pathway for these features may have derived from a bifunctional arabinosidase/xylobiosidase ancestor from which subsites −1 and +1 have acquired new binding capacities and a new subsite +2 occurred in a different orientation. Hence, a detailed analysis of R_09-02, including the resolution of its crystallographic structure by X-ray diffraction analysis, would be of great interest to understand the basis of its peculiar catalytic specificity and thermal characteristics. This information may be valuable for designing protein evolution strategies to modify the substrate specificity of other GHF43 enzymes that have been previously annotated as β-xylosidases, α-xylanases, α-L-arabinases and α-L-arabinofuranosidases in the databases.

The discovery of a novel multifunctional R_09-02 enzyme is a clear example of the utility of function-centred enzyme discovery in complex microbial communities. The natural selection caused by the pressure of the great polymeric substrate diversity imposed on a complex microbial community is likely a key factor that drives the evolution of the conventional GHF43 enzymes. This evolution may have resulted in the modification of enzymes that act on pentose-based polymeric substrates toward the hydrolysis of hexose-containing compounds, conferring a biological advantage for the enzyme-producing organism by expanding its substrate spectrum. Because GHF43 is a highly represented enzyme family in the rumen and many proteins of this family share a high degree of homology with R_09-02, we suggest that the enzymatic potential of the microorganisms in animal GI tracts to degrade plant biomass components that contain arabinose, xylose, galactose and glucose has thus far been underestimated. The present study highlights the need for more extensive and rigorous experimental studies to accurately assess the enzyme activities from (meta-) genomic data.

## Supporting Information

Figure S1 **Physical maps of the r_01, r_02, r_03, r_05, r_06, r_07, r_09 fosmid/plasmid from the R library.**
(PDF)

Figure S2 **Temperature optima for the hydrolases recovered from the R library.** The enzyme activity was determined as described in the Supporting Materials and Methods using the best substrate and pH (see the details in **Table S5**) and the enzyme at a concentration of 12 nM.
(PDF)

Figure S3 **pH optima for the hydrolases recovered from the R library.** The enzyme activity was determined as described in the Supporting Materials and Methods using the best substrate and temperature (see the details in **Table S5**) and the enzyme at a concentration of 12 nM.
(PDF)

Figure S4 **Domain organisation of the rumen hydrolases identified in the present work, according to sequence using the Pfam database.** The signal peptides predicted using the SignalP server are indicated with a red dot at the N-terminal site.
(PDF)

Figure S5 **Overall 3-D modelling of the structure of the hydrolases from the R library.** The residues belonging to the catalytic core and regions that are suggested to have functional and structural roles are indicated. The following proteins were used as the templates for the homology modelling: β-galactosidase from *Bacteroides vulgatus* (PDB 3gm8) for R_01-20; α-galactosidase from *Lactobacillus brevis* (PDB 3mi6) for R_01-21; *Klebsiella* sp. isomaltulose synthase and related enzymes (PDB 1wzl, 1wza and 1m53) for R_02-15; α-arabinofuranosidase from *Bacillus subtilis* (PDB 3c7g) for R_03-04, R_03-05 and R09-02; and α-rhamnosidase from *Bacteroides thetaiotaomicron* (PDB 3cih) for R_07-01 and R_08-01.
(PDF)

Figure S6 **R_09-02, as overexpressed in the active form in** ***E. coli*** **at low temperatures.** The quantification of the activity level (**A**) and optical density (**B**) of cells expressing R_09-02 was performed at 37, 28 and 22 °C at the indicated time points. Please refer to the Materials and Methods for details of the activity quantification (using *p*NPβX as the substrate). (**C**, **D**) A Coomassie-stained SDS**-**PAGE gel showing the purification of the R_09-02 protein. Only R_09-02, which represents the most atypical enzyme in terms of its biochemical characteristics, is shown; the other enzymes derived from the R library were also found to be more than 98% pure (data not shown). (**C**) SDS-PAGE gel showing the gene expression at 37°C and the presence of inclusion bodies. (**D**) SDS-PAGE gel showing the gene expression at 20°C. As shown, a high percentage of protein is produced in a soluble form, which resulted in a purity higher than 98% after a single His_6_-tag purification step.
(PDF)

Figure S7 **Dendrogram of the compositional sequence similarities, as calculated by the comparison of the frequencies of tetranucleotides in the sequenced DNA fragments, of the r_02 fosmid and bacterial chromosomes.**
(PDF)

Figure S8 **General structures of the activated and non-activated oligosaccharide substrates for R_09-02.** The arrow indicates the putative cleavage site.
(PDF)

Table S1 **Summary of the characteristics of selected fosmid/plasmid clones from the bovine rumen (R) metagenome library that contains genes encoding glycosyl hydrolases.**
(PDF)

Table S2 **Annotation of the genes predicted in the fosmid/plasmid clones from the bovine rumen (R) metagenome library. (A) Fosmid r_01, (B) fosmid r_02, (C) fosmid r_03, (D) plasmid r_05, (E) plasmid r_06, (F) plasmid r_07, (G) plasmid r_08 and (H) plasmid r_09.** Selected fosmids were sequenced by shotgun sequencing, and the sorted ORFs were annotated by homology using the BLAST alignment tool. The theoretical molecular weight (MW) and isoelectric point (p*I*) were calculated for each gene product using the ExPASy ProtParam online tool.
(PDF)

Table S3 **Summary of the annotation features of the glycosyl- and feruloyl-like coding sequences (CDSs) predicted in the hydrolase-coding DNA fragments from the R library.**
(PDF)

Table S4 **Summary of the annotation features of the carbohydrate accessory enzymes identified in the hydrolase-encoding DNA fragments from the R library.**
(PDF)

Table S5 **Kinetic parameters of the glycosyl and feruloyl hydrolases that were subcloned, expressed, purified and characterised in this study.**
(PDF)

Table S6 **Vectors (A) and oligonucleotides (B) used in this work.**
(PDF)

Table S7 **Biochemical information of GHF43 enzymes described in** **Figure 2** **. Data are based on bibliographic records that are specifically cited.**
(PDF)

Methods S1 **Complete description of materials and methods and cloning, expression and purification of the plant polymeric-substance hydrolases.**
(DOC)

Text S1 **Complete description of rumen degradative enzymes (phylogeny and biochemistry), analysis of the DNA fragments using genome linguistics and 3-D modelling analysis of microbial hydrolases from the R library.**
(DOC)

References S1 **Complete list of citations for Methods S1 and Text S1.**
(DOC)
