# Supplementary figures and images for: Functional Metagenomics Unveils a Multifunctional Glycosyl Hydrolase from the Family 43 Catalysing the Breakdown of Plant Polymers in the Calf Rumen
Source: PLoS One. 2012 Jun 25;7(6):e38134. doi: 10.1371/journal.pone.0038134 (PMC3382598; doi:10.1371/journal.pone.0038134)

Figure S1 Physical maps of the r\_01, r\_02, r\_03, r\_05, r\_06, r\_07, r\_09 fosmids/plasmids from the R library.

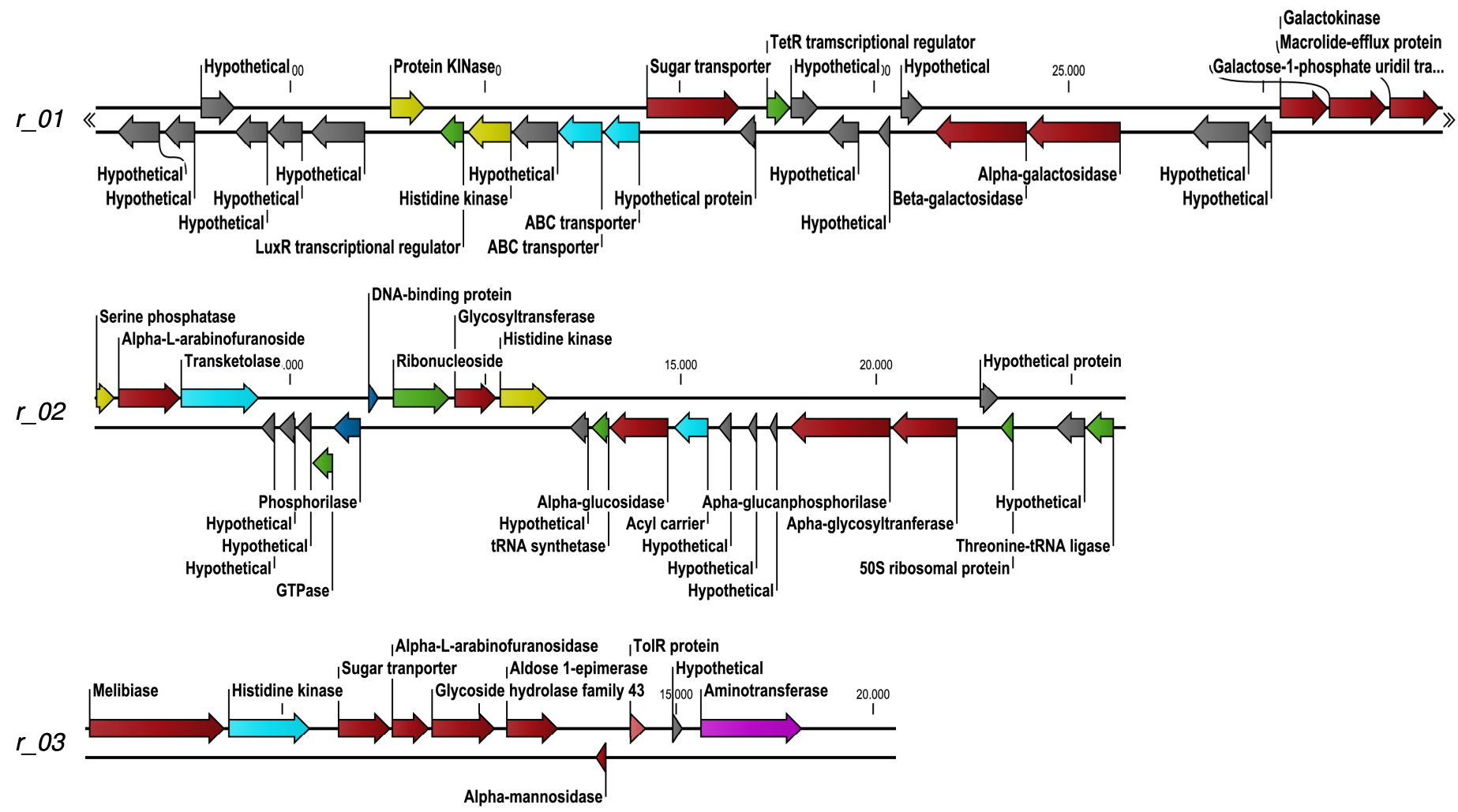

(B) Plasmids r\_05, r\_06, r\_07 and r\_09

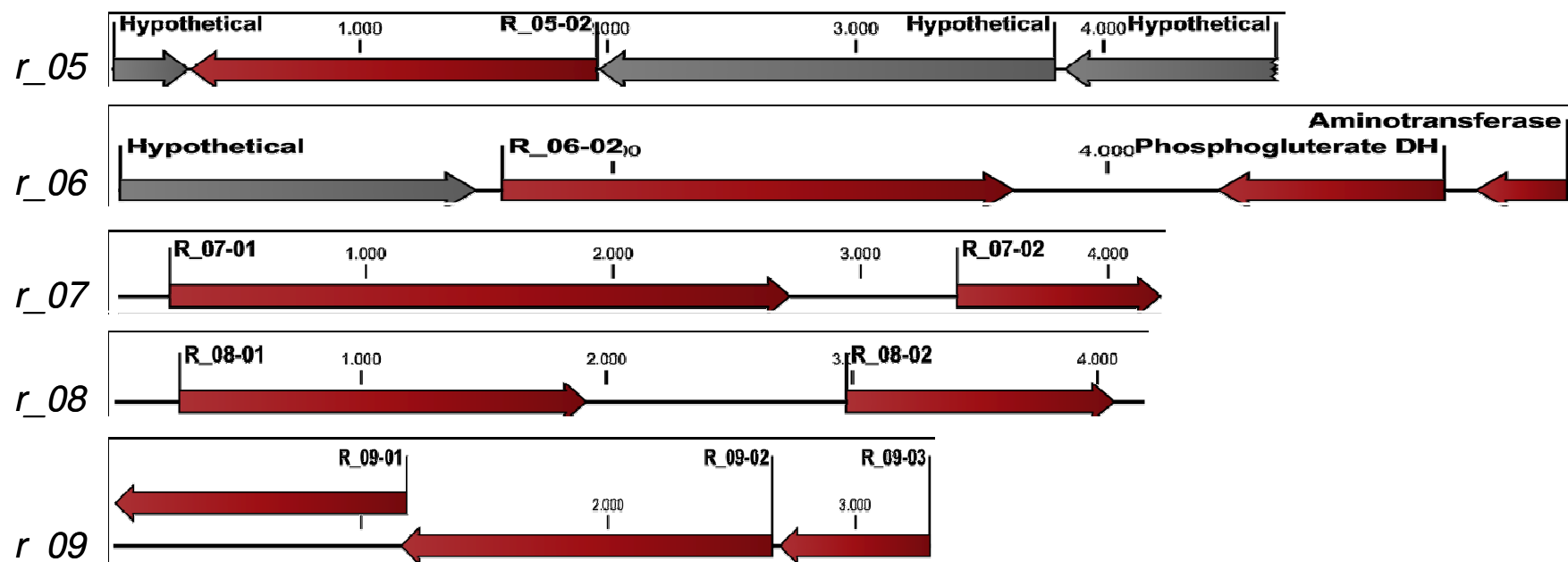

Supplement: Figure S1 — Physical maps of the r_01, r_02, r_03, r_05, r_06, r_07, r_09 fosmid/plasmid from the R library. (PDF) [file pone.0038134.s001.pdf]
