# Supplementary material for: Functional Metagenomics Unveils a Multifunctional Glycosyl Hydrolase from the Family 43 Catalysing the Breakdown of Plant Polymers in the Calf Rumen
Source: PLoS One. 2012 Jun 25;7(6):e38134. doi: 10.1371/journal.pone.0038134 (PMC3382598; doi:10.1371/journal.pone.0038134)

Figure S2 Temperature optima for the hydrolases recovered from the R library. The enzyme activity was determined as described in the Supporting Materials and Methods using the best substrate and pH (see the details in **Table S5**) and the enzyme at a concentration of 12 nM.

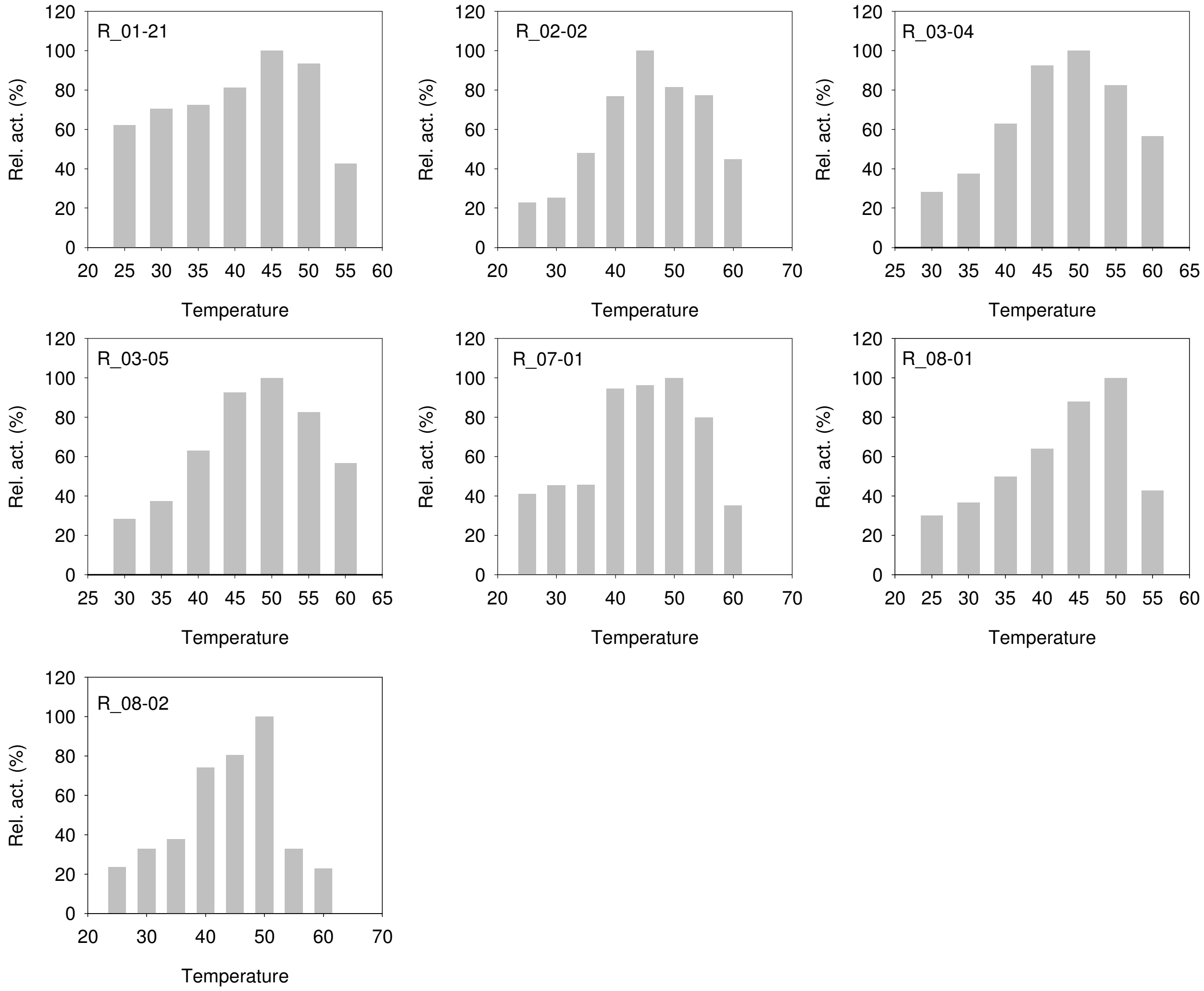

Supplement: Figure S2 — Temperature optima for the hydrolases recovered from the R library. The enzyme activity was determined as described in the Supporting Materials and Methods using the best substrate and pH (see the details in Table S5) and the enzyme at a concentration of 12 nM. (PDF) [file pone.0038134.s002.pdf]
