# Supplementary material for: Functional Metagenomics Unveils a Multifunctional Glycosyl Hydrolase from the Family 43 Catalysing the Breakdown of Plant Polymers in the Calf Rumen
Source: PLoS One. 2012 Jun 25;7(6):e38134. doi: 10.1371/journal.pone.0038134 (PMC3382598; doi:10.1371/journal.pone.0038134)

Figure S4 Domain organisation of the rumen hydrolases identified in the present work, according to sequence using the Pfam database. The signal peptides predicted using the SignalP server are indicated with a red dot at the N-terminal site.

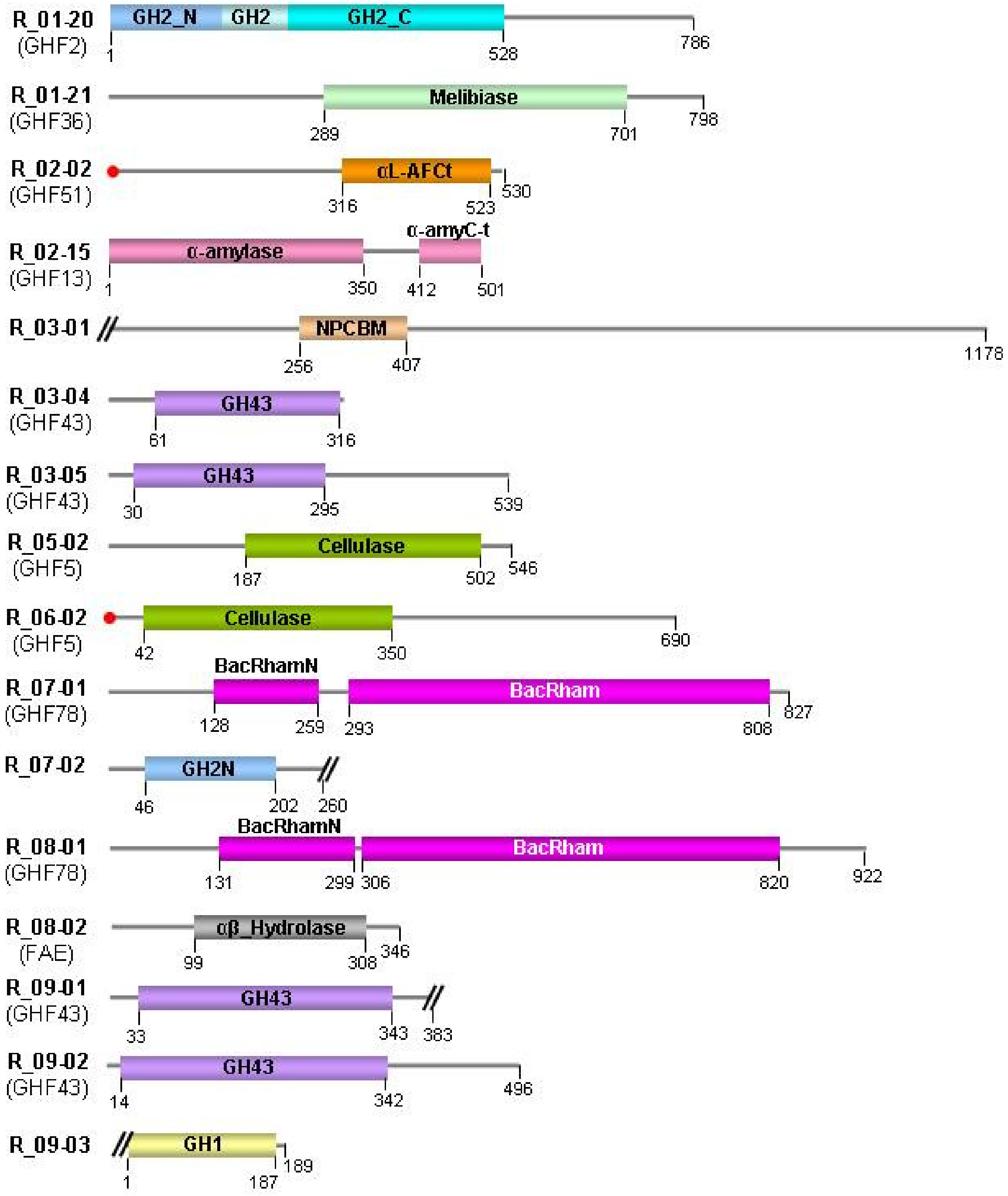

Supplement: Figure S4 — Domain organisation of the rumen hydrolases identified in the present work, according to sequence using the Pfam database. The signal peptides predicted using the SignalP server are indicated with a red dot at the N-terminal site. (PDF) [file pone.0038134.s004.pdf]
