# Supplementary material for: Functional Metagenomics Unveils a Multifunctional Glycosyl Hydrolase from the Family 43 Catalysing the Breakdown of Plant Polymers in the Calf Rumen
Source: PLoS One. 2012 Jun 25;7(6):e38134. doi: 10.1371/journal.pone.0038134 (PMC3382598; doi:10.1371/journal.pone.0038134)

Figure S5 Overall 3-D modelling of the structure of the hydrolases from the R library. The residues belonging to the catalytic core and regions that are suggested to have functional and structural roles are indicated. The following proteins were used as the templates for the homology modelling: β-galactosidase from *Bacteroides vulgatus* (PDB 3gm8) for R_01-20; α-galactosidase from *Lactobacillus brevis* (PDB 3mi6) for R_01-21; *Klebsiella* sp. isomaltulose synthase and related enzymes (PDB 1wzl, 1wza and 1m53) for R_02-15; α-arabinofuranosidase from *Bacillus subtilis* (PDB 3c7g) for R_03-04, R_03-05 and R09-02; and α-rhamnosidase from *Bacteroides thetaiotaomicron* (PDB 3cih) for R_07-01 and R_08-01.

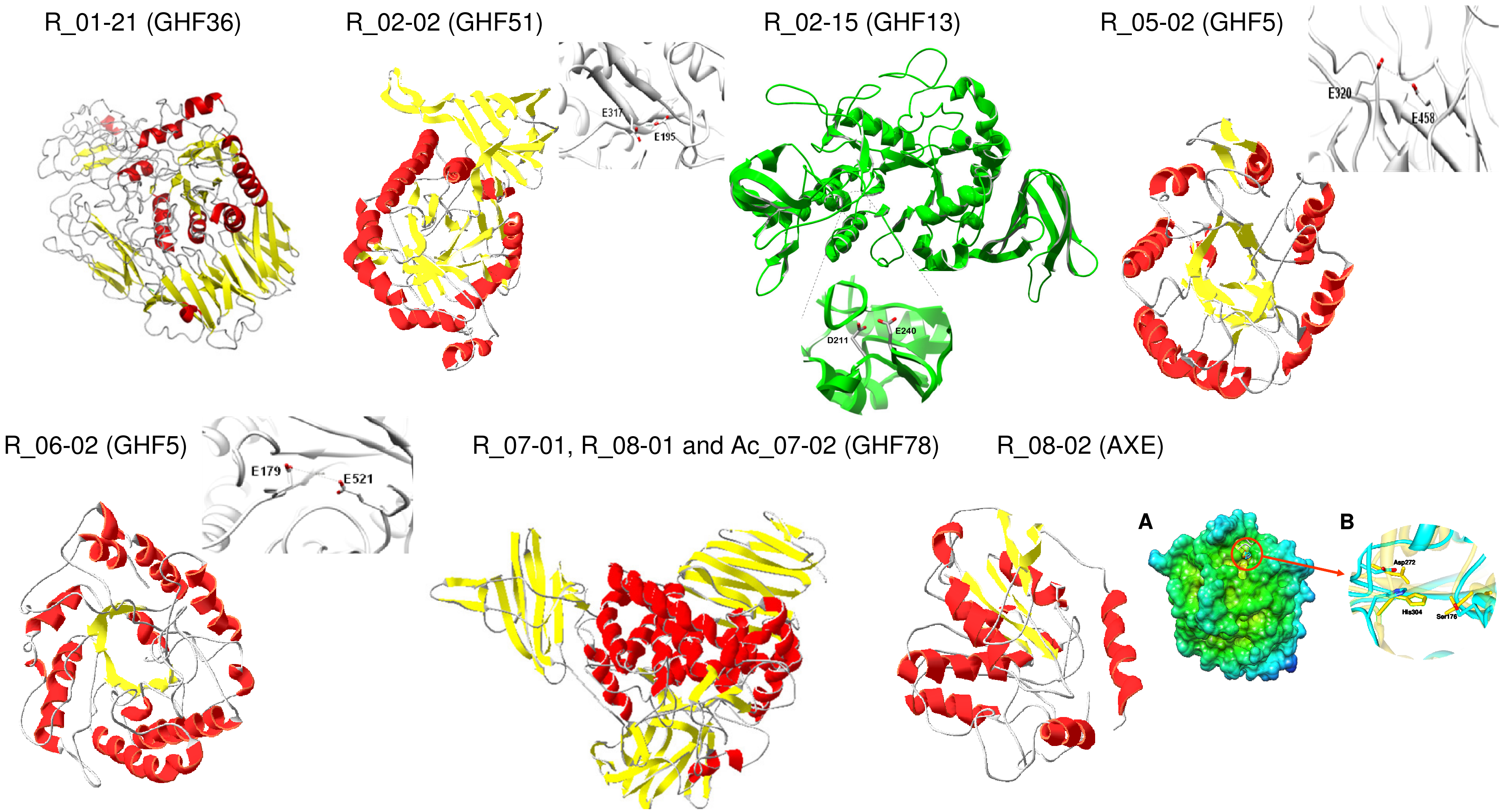

Supplement: Figure S5 — Overall 3-D modelling of the structure of the hydrolases from the R library. The residues belonging to the catalytic core and regions that are suggested to have functional and structural roles are indicated. The following proteins were used as the templates for the homology modelling: β-galactosidase from Bacteroides vulgatus (PDB 3gm8) for R_01-20; α-galactosidase from Lactobacillus brevis (PDB 3mi6) for R_01-21; Klebsiella sp. isomaltulose synthase and related enzymes (PDB 1wzl, 1wza and 1m53) for R_02-15; α-arabinofuranosidase from Bacillus subtilis (PDB 3c7g) for R_03-04, R_03-05 and R09-02; and α-rhamnosidase from Bacteroides thetaiotaomicron (PDB 3cih) for R_07-01 and R_08-01. (PDF) [file pone.0038134.s005.pdf]
