# Supplementary material for: Functional Metagenomics Unveils a Multifunctional Glycosyl Hydrolase from the Family 43 Catalysing the Breakdown of Plant Polymers in the Calf Rumen
Source: PLoS One. 2012 Jun 25;7(6):e38134. doi: 10.1371/journal.pone.0038134 (PMC3382598; doi:10.1371/journal.pone.0038134)

Figure S6 R_09-02, as overexpressed in the active form in *E. coli* at low temperatures. The quantification of the activity level **(A)** and optical density **(B)** of cells expressing R_09-02 was performed at 37, 28 and 22 ºC at the indicated time points. Please refer to the Materials and Methods for details of the activity quantification (using *p*NPβX as the substrate). **(C, D)** A Coomassie-stained SDS-PAGE gel showing the purification of the R_09-02 protein. Only R_09-02, which represents the most atypical enzyme in terms of its biochemical characteristics, is shown; the other enzymes derived from the R library were also found to be more than 98% pure (data not shown). **(C)** SDS-PAGE gel showing the gene expression at 37 ºC and the presence of inclusion bodies. **(D)** SDS-PAGE gel showing the gene expression at 20 ºC. As shown, a high percentage of protein is produced in a soluble form, which resulted in a purity higher than 98% after a single His_6-tag purification step.

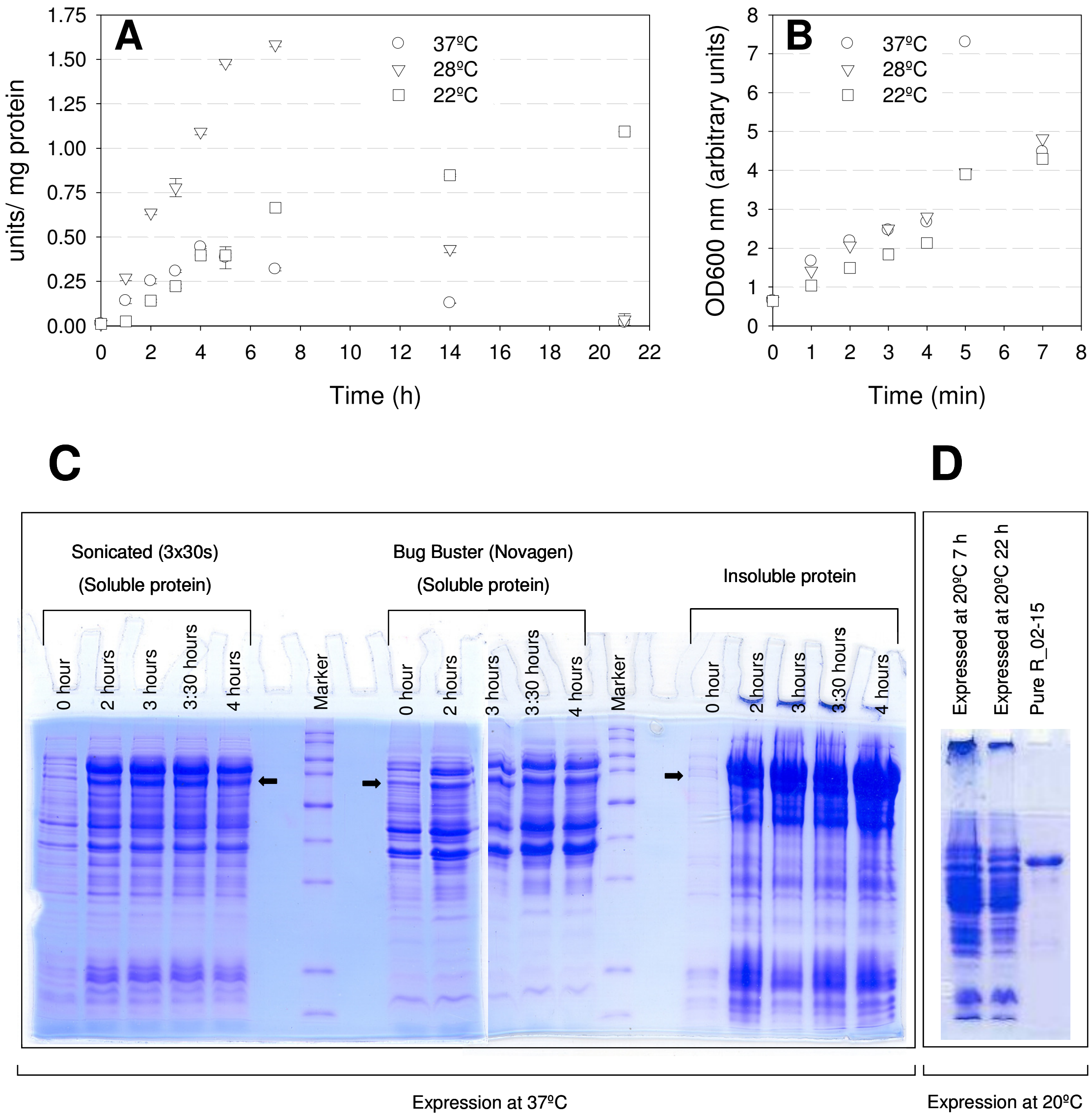

Supplement: Figure S6 — R_09-02, as overexpressed in the active form in E. coli at low temperatures. The quantification of the activity level (A) and optical density (B) of cells expressing R_09-02 was performed at 37, 28 and 22 °C at the indicated time points. Please refer to the Materials and Methods for details of the activity quantification (using pNPβX as the substrate). (C, D) A Coomassie-stained SDS-PAGE gel showing the purification of the R_09-02 protein. Only R_09-02, which represents the most atypical enzyme in terms of its biochemical characteristics, is shown; the other enzymes derived from the R library were also found to be more than 98% pure (data not shown). (C) SDS-PAGE gel showing the gene expression at 37°C and the presence of inclusion bodies. (D) SDS-PAGE gel showing the gene expression at 20°C. As shown, a high percentage of protein is produced in a soluble form, which resulted in a purity higher than 98% after a single His6-tag purification step. (PDF) [file pone.0038134.s006.pdf]
