# Supplementary material for: Functional Metagenomics Unveils a Multifunctional Glycosyl Hydrolase from the Family 43 Catalysing the Breakdown of Plant Polymers in the Calf Rumen
Source: PLoS One. 2012 Jun 25;7(6):e38134. doi: 10.1371/journal.pone.0038134 (PMC3382598; doi:10.1371/journal.pone.0038134)

Figure S7 Dendrogram of the compositional sequence similarities, as calculated by the comparison of the frequencies of tetranucleotides in the sequenced DNA fragments, of the r_02 fosmid and bacterial chromosomes.

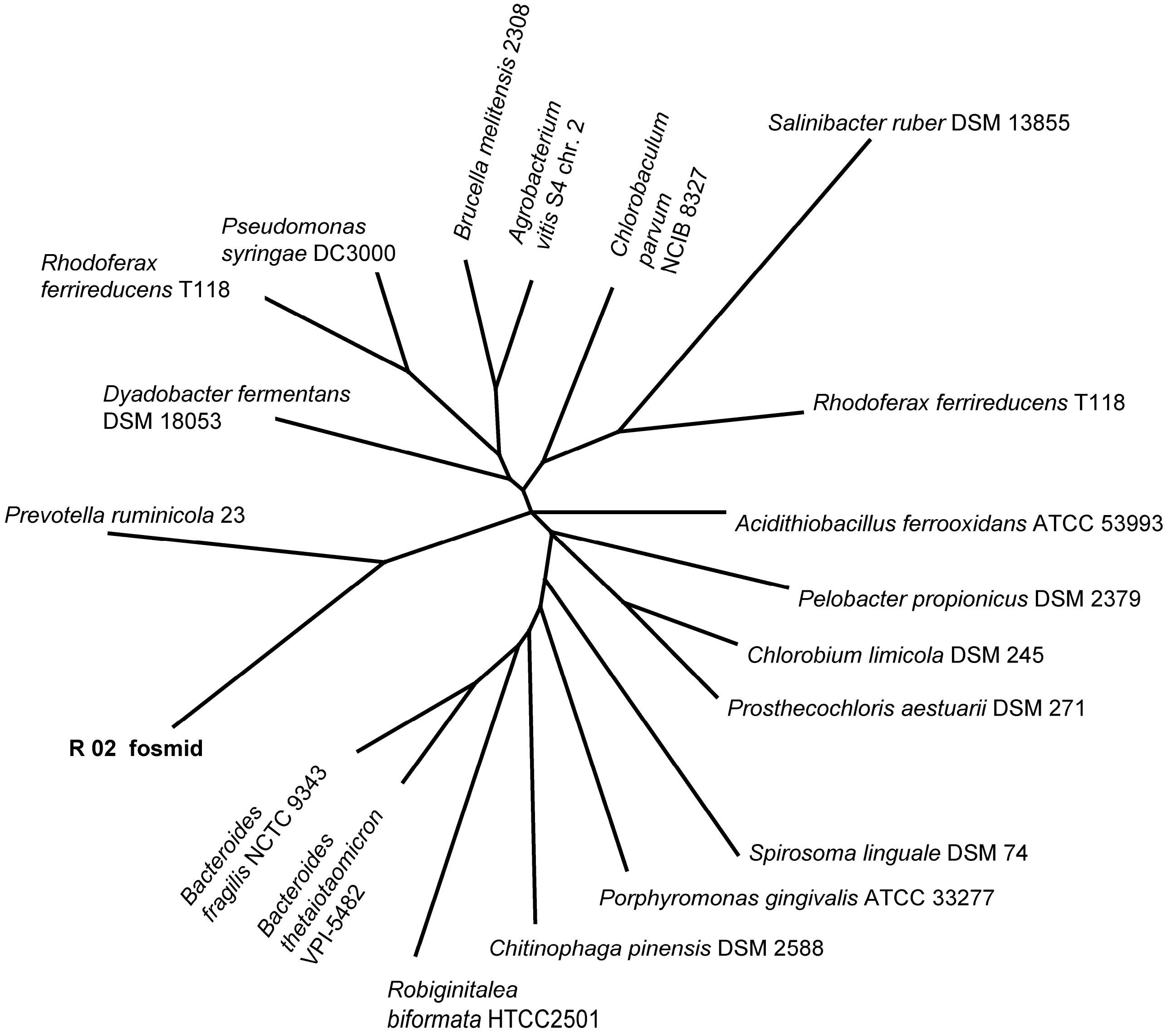

Supplement: Figure S7 — Dendrogram of the compositional sequence similarities, as calculated by the comparison of the frequencies of tetranucleotides in the sequenced DNA fragments, of the r_02 fosmid and bacterial chromosomes. (PDF) [file pone.0038134.s007.pdf]
