# Supplementary material for: Functional Metagenomics Unveils a Multifunctional Glycosyl Hydrolase from the Family 43 Catalysing the Breakdown of Plant Polymers in the Calf Rumen
Source: PLoS One. 2012 Jun 25;7(6):e38134. doi: 10.1371/journal.pone.0038134 (PMC3382598; doi:10.1371/journal.pone.0038134)

Figure S8 General structures of the activated and non-activated oligosaccharide substrates for R_09-02. The arrow indicates the putative cleavage site.

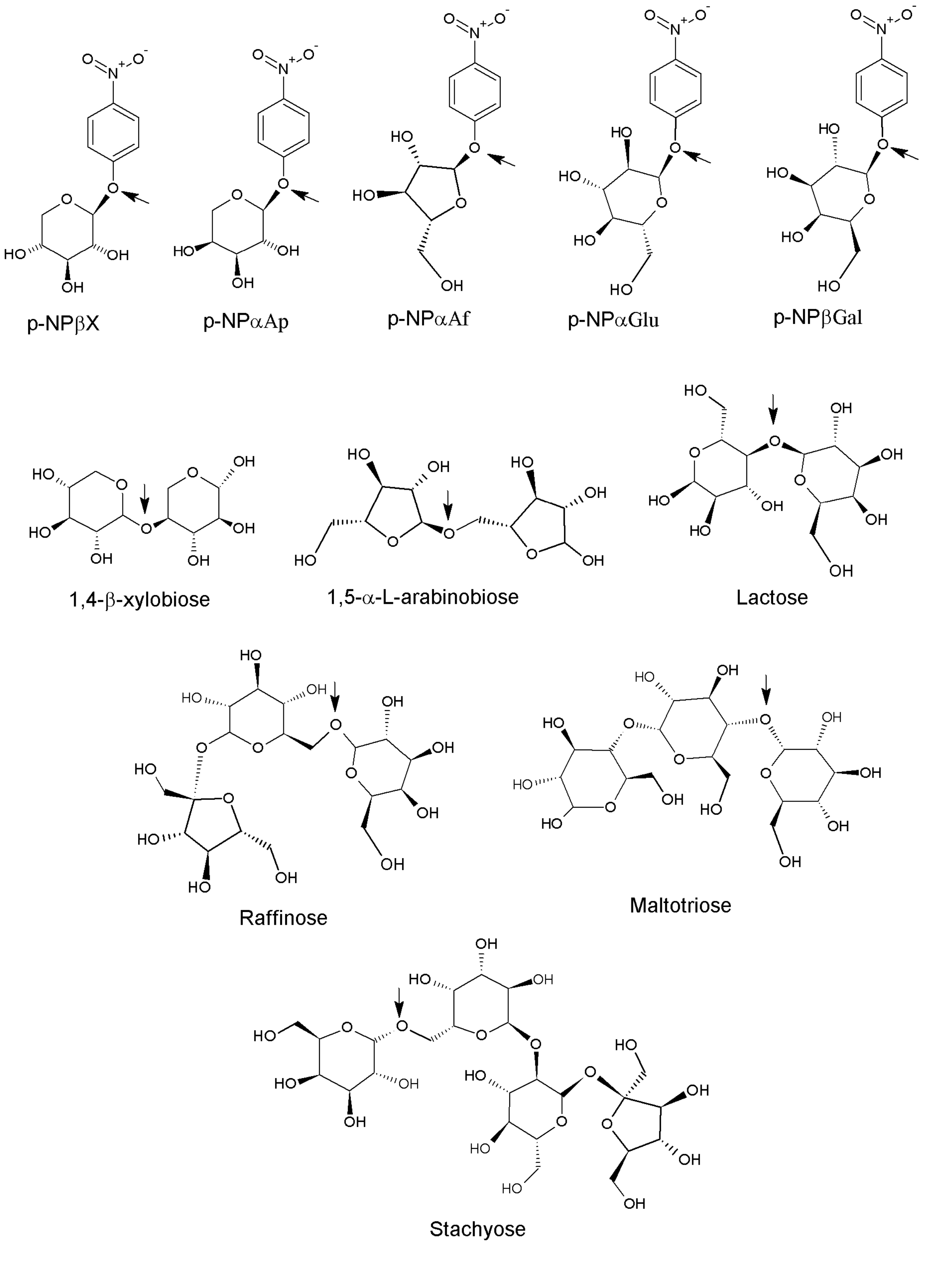

Supplement: Figure S8 — General structures of the activated and non-activated oligosaccharide substrates for R_09-02. The arrow indicates the putative cleavage site. (PDF) [file pone.0038134.s008.pdf]
