# Supplementary material for: Functional Metagenomics Unveils a Multifunctional Glycosyl Hydrolase from the Family 43 Catalysing the Breakdown of Plant Polymers in the Calf Rumen
Source: PLoS One. 2012 Jun 25;7(6):e38134. doi: 10.1371/journal.pone.0038134 (PMC3382598; doi:10.1371/journal.pone.0038134)
Supplement: Table S1 — Summary of the characteristics of selected fosmid/plasmid clones from the bovine rumen (R) metagenome library that contains genes encoding glycosyl hydrolases. (PDF) [file pone.0038134.s009.pdf]

Table S1 Summary of the characteristics of selected fosmid/plasmid clones from the bovine rumen (R) metagenome library that contain genes encoding glycosyl hydrolases.

Fosmid/ plasmid designation	Source	Screened substrate	Vector	Size (bp)	G+C average (%)	Nº ORFs	Nº GH
r_01	Cow rumen	pNPαGal	pCC1FOS	34609	53.78	27	2
r_02	Cow rumen	pNPαAf	pCC1FOS	26383	50.89	25	2
r_03	Cow rumen	pNPαAf	pCC1FOS	19873	62,86	10	2
r_05	Cow rumen	CMC	pUC19	4638	51,53	4	1
r_06	Cow rumen	CMC	pUC19	5805	44,60	4	1
r_07	Cow rumen	pNPαR	pUC19	4176	63,27	2	2
r_08	Cow rumen	pNPαR	pUC19	4145	63,86	2	1
r_09	Cow rumen	pNPαAf	pUC19	3264	60,69	3	3
