# Supplementary material for: Functional Metagenomics Unveils a Multifunctional Glycosyl Hydrolase from the Family 43 Catalysing the Breakdown of Plant Polymers in the Calf Rumen
Source: PLoS One. 2012 Jun 25;7(6):e38134. doi: 10.1371/journal.pone.0038134 (PMC3382598; doi:10.1371/journal.pone.0038134)
Supplement: Table S2 — Annotation of the genes predicted in the fosmid/plasmid clones from the bovine rumen (R) metagenome library. (A) Fosmid r_01, (B) fosmid r_02, (C) fosmid r_03, (D) plasmid r_05, (E) plasmid r_06, (F) plasmid r_07, (G) plasmid r_08 and (H) plasmid r_09. Selected fosmids were sequenced by shotgun sequencing, and the sorted ORFs were annotated by homology using the BLAST alignment tool. The theoretical molecular weight (MW) and isoelectric point (pI) were calculated for each gene product using the ExPASy ProtParam online tool. (PDF) [file pone.0038134.s010.pdf]

Table S2 Annotation of the genes predicted in the fosmid/plasmid clones from the bovine rumen (R) metagenome library. (A) Fosmid r_01, (B) fosmid r_02, (C) fosmid r_03, (D) plasmid r_05, (E) plasmid r_06, (F) plasmid r_07, (G) plasmid r_08 and (H) plasmid r_09. Selected fosmids were sequenced by shotgun sequencing, and the sorted ORFs were annotated by homology using the BLAST alignment tool. The theoretical molecular weight (MW) and isoelectric point (p*I*) were calculated for each gene product using the ExPASy ProtParam online tool.

(A) Fosmid *r_01*

Gene name	Strand	Left End	Right End	Length (AA)	Gene product name	MW (Da)*	pI*	Organism (best hit)	Expected Value (E), Identities (I), Positives (P)	Top homolog
r_01-01	-	554	1651	365	Hypothetical protein	41519.62	6.55	*Coprococcus sp.*	E=4e-44; I=118/389 (30%); P=188/389 (48%)	CBK83438.1
r_01_02	-	1756	2556	266	Hypothetical protein	29042.66	4.26	*Eubacterium hallii*	E=2e-38; I=83/213 (38%); P=122/213 (57%)	ZP_03717785.1
r_01_03	+	2691	3578	295	Hhypothetical protein	32939.75	4.2	*Desulfovibrio piger*	E=4e-72; I=143/294 (48%); P=186/294 (63%)	ZP_03311848.1
r_01_04	-	3575	4423	282	Hypothetical protein	32109.03	5.88	*Eubacterium yurii*	E=0.007; I=17/43 (40%), P = 28/43 (66%)	ZP_07454699.1
r_01_05	-	4434	5318	294	Hypothetical protein	33207.73	5.4	*Eubacterium siraeum*	E=0.002; I=19/43 (44%); P=25/43 (58%)	CBK97501.1
r_01_06	-	5521	6924	467	Hhypothetical protein	50863.10	9.01	*Shuttleworthia satelles*	E=3e-103; I=201/463 (43%); P= 298/463 (64%)	ZP_04454784.1
r_01_07	+	7555	8472	305	Pprotein KINase family	34487.97	4.78	*Myxobacter sp.*	E=9e-13; I=71/240 (29%); P=110/240 (45%)	AAX82624.1
r_01_08	-	8835	9464	209	LuxR transcriptional regulator	23070.64	4.52	*Ruminococcus obeum*	E=1e-59; I=114/205(55%); P= 154/205 (75%)	ZP_01964629.1
r_01_09	-	9551	10687	378	Histidine kinase	41561.16	6.48	*Ruminococcus albus*	E=2e-47; I=96/225 (42%); P=145/225 (64%)	ZP_06717737.1
r_01_10	-	10700	11884	394	Hypothetical protein	43208.82	5.21	*Dorea longicatena*	E=2e-29; I=119/401 (29%); P=196/401 (48%)	ZP_01995410.1
r_01_11	-	11871	13019	382	ABC-2 type transporter	42561.68	4.75	*Ruminococcus albus 7*	E=3e-36; I=105/382 (28%); P=189/382 (50%)	ZP_07383825.1
r_01_12	-	13041	13982	313	ABC transporter related	34795.30	5.71	*Ruminococcus albus 7*	E=7e-115; I=205/310 (67%); P=245/310 (80%)	ZP_07383824.1
r_01_13	+	14147	16546	799	Sugar transporter	89500.04	4.02	*Bacillus mycoides*	E=2e-16; I=111/449 (24%); P=198/449 (44%)	ZP_04160063.1
r_01_14	-	16527	16970	147	Hypothetical protein	16164.02	9.36	*Nostoc punctiforme*	E=0.84; I=19/58 (32%; P=31/58 (53%)	YP_001867131.1
r_01_15	+	17233	17850	205	TetR transcriptional regulator	24154.64	5.82	*Clostridium hylemonae*	E=7e-46; I=91/204 (44%); P=137/204 (67%)	ZP_03777844.1
r_01_16	+	17850	18569	239	Hypothetical protein	27040.49	9.45	*Clostridium hylemonae*	E=7e-85; I=145/237 (61%); P=182/237 (76%)	ZP_03778041.1
r_01_17	-	18808	19617	269	Hypothetical protein	31513.88	8.71	*Butyrivibrio fibrisolvens*	E=0.038; I=29/106 (27%); P=53/106 (50%)	CBK73816.1
r_01_18	-	20087	20416	109	Hypothetical protein	12288.62	8.05	*Toxoplasma gondii*	E=3.7; I=22/66 (33%); P=36/66 (54%)	EEE34841.1
r_01_19	+	20677	21270	197	Hypothetical protein	20864.44	4.85	*Listeria welshimeri*	E=0.46; I=35/154 (22%); P=65/154 (42%)	YP_849623.1
r_01_20	**-**	**21563**	**23923**	**786**	**beta-Galactosidase (GHF2)**	**87991.29**	**5.17**	***Roseburia inulinivorans***	**E=0.0; I=441/777 (56%); P=541/777 (69%)**	**ZP_03752805.1**
r_01_21	**-**	**23943**	**26339**	**798**	**alpha-Galactosidase (GHF36)**	**90107.58**	**5.71**	***Roseburia inulinivorans***	**E=0.0; I=482/807 (59%); P=603/807 (74%)**	**ZP_03752801.1**
r_01_22	-	26345	28159	604	Sugar transporter	65605.7	5.14	*Clostridium lentocvellum* DSM 5427	E=1e-86; I=172/299 (58%); P=220/299 (74%)	ZP_06885281.1
r_01_23	-	28175	29641	488	Hypothetical protein	54586.33	6.94	*Roseburia intestinales* L1-82	E=0.0; I=361/488 (73%); P=411/488 (84%)	ZP_04742652.2
r_01_24	-	29658	30224	188	Hypothetical protein	22227.74	5.83	*Roseburia inulinivorans*	E=1e-51; I=99/183 (54%); P=133/183 (72%)	ZP_03752806.1
r_01_25	+	30423	31670	415	Galactokinase	44143.84	4.93	*Clostridium sp.*	E=7e-139; I=240/405 (59%); P=307/405 (75%)	ZP_02073770.1
r_01_26	+	31682	33166	494	Galactose-1-phosphate uridylyltransferase	55737.38	5.4	*Clostridium sp.*	E=0.0; I=315/498 (63%); P=372/498 (74%)	ZP_02073769.1
r_01_27	+	33237	34520	427	Sugar transporter	45007.41	9.13	*Oribacterium sp*	E=3e-117; I=215/423 (50%); P=298/423 (70%)	ZP_06598814.1

*The theoretical molecular weight (MW) and isoelectric point (pI) were calculated for the gene product using ExPASy ProtParam online tool

Table S2 (B) Fosmid *r_02*

Gene name	Strand	Left End	Right End	Length (AA)	Gene product name	MW (Da)	pI	Organism (best hit)	Expected Value (E). Identities (I). Positives (P)	Top homolog
r_02-01	+	31	516	161	Serine phosphatase	17971.32	4.54	*Prevotella ruminicola*	E=3e-47; I=90/152 (59%); P=113/152 (74%)	YP_003576084.1
r_02-02	**+**	**599**	**2191**	**530**	**alpha-L-arabinofuranosidase (GHF51)**	**59485.56**	**6.25**	***Prevotella ruminicola***	**E=0.0; I=458/530 (86%); P=490/530 (92%)**	**YP_003573794.1**
r_02-03	+	2199	4211	670	Transketolase	73006.93	5.31	*Prevotella ruminicola*	E=0.0; I=634/664 (95%); P=649/664 (97%)	YP_003573769.1
r_02-04	-	4259	4618	119	Hypothetical protein	12711.51	4.71	*Anaerococcus vaginalis*	E=0.052; I=23/79 (29%); P=37/79 (46%)	ZP_05471839.1
r_02-05	-	4703	5143	146	Hypothetical protein	*16867.94*	*9.07*	*Prevotella sp*	E=0.002; I= 33/130 (25%); P= 61/130 (46%)	ZP_06406453.1
r_02-06	-	5167	5550	127	Hypothetical protein	15298.50	4.37	*Bacteroides plebeius*	E=7e-47; I=84/124 (67%); P=103/124 (83%)	ZP_03208046.1
r_02-07	-	5570	6103	177	tRNA modification GTPase	20051.82	4.49	*Prevotella ruminicola*	E= 5e-48; I= 94/173 (54%); P= 132/173 (76%)	YP_003575005.1
r_02-08	-	6100	6813	237	Purine nucleoside phosphorylase	25718.42	5.45	*Epulopiscium sp.*	E=4e-89; I=157/232 (67%); P=186/232 (80%)	ZP_02693126.1
r_02-09	+	6998	7273	91	DNA-binding protein HU	10088.72	9.65	*Prevotella melaninogenica*	E=4e-31; I=67/91 (73%); P=76/91 (83%)	YP_003574043.1
r_02-10	+	7627	9087	486	Ribonuclease G	55414.48	9.28	*Prevotella ruminicola*	E=0.0; I=432/480 (90%); P=458/480 (95%)	YP_003574042.1
r_02-11	+	9203	10285	360	Glycosyltransferase	41162.42	8.23	*Prevotella ruminicola*	E= 3e-169; I= 276/360 (76%); P= 320/360 (88%)	YP_003574041.1
r_02-12	+	10360	11607	415	Histidine kinase	47176.97	5.21	*Prevotella ruminicola*	E= 4e-172; I= 291/409 (71%); P= 346/409 (84%)	YP_003574040.1
r_02-13	-	12157	12645	162	Hypothetical protein	16987.36	4.44	*Anopheles gambiae* str. PEST	E=0.18; I= 17/46 (37%); P= 29/46 (64%)	XP_317568.3
r_02-14	-	12705	13172	155	Alanyl-tRNA synthetase	18340.74	5.31	*Prevotella ruminicola*	E=8e-69; I=126/152 (82%); P=144/152 (94%)	YP_003574038.1
r_02-15	**-**	**13177**	**14682**	**501**	**alpha-Glucosidase (alpha-amylase) (GHF13)**	**56950.34**	**5.53**	***Prevotella ruminicola***	**E=0.0; I= 398/501 (79%); P= 436/501 (87%)**	**YP_003574036.1**
r_02-16	-	14819	15709	296	Malonyl CoA-acyl carrier protein	31591.42	5.04	*Prevotella ruminicola*	E=2e-164; I=283/296 (95%); P=289/296 (97%)	YP_003574035.1
r_02-17	-	15959	16300	113	Hypothetical protein	12599.67	8.51	*Trichoplax adhaerens*	E=0.45; I=22/57 (38%); P=32/57 (56%)	XP_002109933.1
r_02-18	-	16713	16955	80	Hypothetical protein	8357.08	4.6	*Ostreococcus tauri*	E=0.042; I=26/80 (33%); P=40/80 (50%)	XP_003074850.1
r_02-19	-	17262	17477	71	Hypothetical protein	7465.39	4.87	*Phaeodactylum tricornutum*	E=7.5; I=16/37 (43%); P=21/37 (56%)	XP_002177766.1
r_02-20	-	17792	20368	858	alpha-Glucan phosphorylase	99019.84	6	*Prevotella ruminicola*	E=0.0; I=766/856 (89%); P=819/856 (95%)	YP_003574031.1
r_02-21	-	20391	22082	563	alpha-Glycosyltransferase	64617.55	5.55	*Prevotella ruminicola*	E=0.0; I=466/576 (80%); P=503/576 (87%)	YP_003574030.1
r_02-22	+	22645	23139	164	Hypothetical protein	17633.17	8.14	*Prevotella ruminicola*	E=3e-77; I=140/157 (89%); P=152/157 (96%)	YP_003575276.1
r_02-23	-	23174	23518	114	50S ribosomal protein L20	13155.52	11.25	*Prevotella ruminicola*	E=4e-57; I=111/114 (97%); P=111/114 (97%)	YP_003573965.1
r_02-24	-	24594	25349	251	Hypothetical protein	28037.20	6.53	*Branchiostoma floridae*	E=4e-09; I=38/148 (25%); P=71/148 (47%)	XP_002611623.1
r_02-25	-	25357	26085	242	Threonine-tRNA ligase	27421.26	5.1	*Prevotella ruminicola*	E=5e-138; I=236/242 (97%); P=240/242 (99%)	YP_003573962.1

*The theoretical molecular weight (MW) and isoelectric point (pI) were calculated for the gene product using ExPASy ProtParam online tool

Table S2 (C) Fosmid *r_03*

Gene name	Strand	Left End	Right End	Length (AA)	Gene product name	MW (Da)	pI	Organism (best hit)	Expected Value (E). Identities (I). Positives (P)	Top homolog
r_03-01	+	1	3540	1148	Melibiase (CBM51)	119432.86	4.48	*Blastopirellula marina*	E=3e-06; I=43/150 (28%); P=77/150 (51%)	ZP_01089974.1
r_03-02	+	3626	5707	693	Histidine kinase	74948.66	5.96	*Chthononiobacter flavus*	E=2e-37; I=160/610 (26%); P=257/610 (42%)	ZP_03131882.1
r_03-03	+	6411	7748	445	Sugar transporter	47558.78	9.21	*Bacteriodes xylanisolvens*	E=2e-147; I=267/486 (54%); P=335/486 (68%)	CBK69517.1
r_03-04	**+**	**7778**	**8731**	**317**	**beta-Xylosidase (GHF43)**	**35572.72**	**5.08**	***Bacteroides intestinalis***	**E=2e-102; I=184/316 (58%); P=228/316 (72%)**	**YP_001297392.1**
r_03-05	**+**	**8786**	**10405**	**539**	**alpha-L-Arabino-furanosidase (GHF43)**	**59988.12**	**7.20**	***Bacteroides xylanisolvens***	**E=7e-170; I288/527 (54%=); P=378/527 (71%)**	**ZP_06769240.1**
r_03-06	+	10683	12005	440	Aldose 1-epimerase	48897.69	9.46	*Opitutus terrae*	E=8e-96; I=172/357 (48%); P=228/357 (63%)	YP_001818271.1
r_03-07	-	12951	13232	93	Hypothetical protein	10227.92	11.45	*Arabidopsis lyrata*	E=6.0; I=20/58 (50%); P=29/58 (50%)	XP_002870203.1
r_03-08	+	13817	14239	140	TolR protein	15875.55	9.13	*Francisella tularensis*	E=2e-16; I=44/145 (30%); P=85/145 (58%)	YP_169843.1
r_03-09	+	14894	15172	92	Hypothetical protein	10218.49	11.66	*Rubrobacter xylanophilus*	E=7.3; I=21/49 (42%); P=26/49 (53%)	YP_643483.1
r_03-10	+	15612	18197	861	Aminotransferase	95115.21	11.55	*Desulfovibrio salexigens*	E=3e-87; I=172/369 (46%); P=235/369 (63%)	YP_002993204.1

*The theoretical molecular weight (MW) and isoelectric point (pI) were calculated for the gene product using ExPASy ProtParam online tool

Table S2 (D) Plasmid *r_05*

Gene name	Strand	Left End	Right End	Length (AA)	Gene product name	MW (Da)*	pI	Organism (best hit)	Expected Value (E). Identities (I). Positives (P)	Top homolog
r_05-01	+	<1	312	104	Probable lipoprotein	11631.30	9.25	*Prevotella ruminicola*	E=3e-23; I=46/97 (47%); P=70/97 (72%)	YP_003575494.1
r_05-02	**-**	**309**	**1949**	**546**	**Glycosyl hydrolase family 5 (GHF5)**	**61222.36**	**5.31**	**Unidentified microorganism**	**E=0.0; I=328/518 (63%); P=386/518 (74%)**	**CAJ19140.1**
r_05-03	-	1956	3791	611	Hypothetical protein	65171.72	4.65	*Bacteroides plebeius*	E=3e-48; I=127/360 (35%); P=197/360 (54%)	ZP_03210084.1
r_05-04	-	3831	>4637	267	Hypothetical protein	29364.18	4.57	*Bacteroides cellulosilyticus*	E=4e-54; I=127/293 (43%); P=168/293 (57%)	ZP_03678036.1

*The theoretical molecular weight (MW) and isoelectric point (pI) were calculated for the gene product using ExPASy ProtParam online tool

Table S2 (E) Plasmid *r_06*

Gene name	Strand	Left End	Right End	Length (AA)	Gene product name	MW (Da)*	pI	Organism (best hit)	Expected Value (E). Identities (I). Positives (P)	Top homolog
r_06-01	+	<2	1456	483	Histidine kinase	55673.40	5.33	*Prevotella ruminicola*	E=6e-109; I=221/481 (45%); P=317/481 (65%)	YP_003575472.1
r_06-02	**+**	**1560**	**3632**	**690**	**Endo-1,4-beta-xylanase (GHF5)**	**78164.3**	**4.87**	***Prevotella ruminicola***	**E=5e-142; I=250/403 (62%); P=294/403 (72%)**	**YP_003575492.1**
r_06-03	-	4439	5368	308	D-Phosphoglycerate dehydrogenase	32933.81	5.33	*Prevotella ruminicola*	E=2e-151; I=288/305 (94%); P=296/305 (97%)	YP_003574366.1
r_06-04	-	>5482	5805	106	Phosphoserine transaminase	11834.54	5.71	*Prevotella ruminicola*	E=3e-57; I=105/109 (96%); P=107/109 (98%)	YP_003574368.1

*The theoretical molecular weight (MW) and isoelectric point (pI) were calculated for the gene product using ExPASy ProtParam online tool

Table S2 (F) Plasmid *r_07*

Gene name	Strand	Left End	Right End	Length (AA)	Gene product name	MW (Da)*	pI	Organism (best hit)	Expected Value (E). Identities (I). Positives (P)	Top homolog
r_07-01	+	<2	202	79	Putative glycosyl hydrolase (GHF43)	8947.0	6.46	*Opitutus terrae* PB90-1	E=4e-12; I=34/69 (49%); P=46/69 (67%);	YP_001818251
r_07-02	**+**	**238**	**2721**	**827**	**alfa-L-Rhamnosidase (GHF78)**	**94254.9**	**4.93**	***Clostridium stercorarium***	**E=7e-33; I=352/832 (42%); P=496/832 (59%)**	**CAB53341.1**
r_07-03	+	2718	3560	280	beta-lactamase domain-containing protein	31172.3	5.41	*Clostridium sp. DL-VIII*	E=7e-42; I=92/279 (33%); P=133/279 (48%);	ZP_09205102
r_07-04	**+**	**3436**	**>4176**	**260**	**beta-Galactosidase (GHF2)**	**29543.47**	**8.69**	***Roseburia intestinalis***	**E=0.0; I=87/206 (42%); P=117/206 (56%)**	**CBL10828.1**

*The theoretical molecular weight (MW) and isoelectric point (pI) were calculated for the gene product using ExPASy ProtParam online tool

Table S2 (G) Plasmid *r_08*

Gene name	Strand	Left End	Right End	Length (AA)	Gene product name	MW (Da)*	pI	Organism (best hit)	Expected Value (E). Identities (I). Positives (P)	Top homolog
r_08-01	**+**	**258**	**3027**	**922**	**alpha-L-Rhamnosidase (GHF78)**	**61801.6**	**5.62**	***Enterococcus casseliflavus***	**E=1e-112; I=208/487 (42%); P=300/487 (61%)**	**ZP_05654693.1**
r_08-02	**+**	**3033**	**4073**	**346**	**alpha/beta Hydrolase**	**40359.5**	**4.95**	***Lysinibacillus sphaericus***	**E=7e-42; I=117/306 (38%); P=153/306 (50%)**	**YP_001698978.1**

*The theoretical molecular weight (MW) and isoelectric point (pI) were calculated for the gene product using ExPASy ProtParam online tool

Table S2 (H) Plasmid *r_09*

Gene name	Strand	Left End	Right End	Length (AA)	Gene product name	MW (Da)*	pI	Organism (best hit)	Expected Value (E). Identities (I). Positives (P)	Top homolog
r_09-01	-	<1	1148	381	Glycosyl hydrolase family 43 (GHF43)	44516.1	4.93	*Bryantella formatexigens*	E=3e-115; I=204/383 (53%); P=272/383 (71%)	ZP_05345324.1
r_09-02	-	1157	2649	496	Xylosidase/arabinosidase (GHF43)	54939.62	4.96	*Bacteroides capillosus*	E=4e-120; I=233/502 (46%); P=303/502 (60%)	ZP_02035907.1
r_09-03	-	2691	>3263	204	beta-Galactosidase (GHF1)	22244.69	4.83	*Ruminococcus sp.*	E=2e-54; I=108/192 (56%); P=131/192 (68%)	CBL17177.1

*The theoretical molecular weight (MW) and isoelectric point (pI) were calculated for the gene product using ExPASy ProtParam online tool
