# Supplementary material for: Functional Metagenomics Unveils a Multifunctional Glycosyl Hydrolase from the Family 43 Catalysing the Breakdown of Plant Polymers in the Calf Rumen
Source: PLoS One. 2012 Jun 25;7(6):e38134. doi: 10.1371/journal.pone.0038134 (PMC3382598; doi:10.1371/journal.pone.0038134)
Supplement: Table S3 — Summary of the annotation features of the glycosyl- and feruloyl-like coding sequences (CDSs) predicted in the hydrolase-coding DNA fragments from the R library. (PDF) [file pone.0038134.s011.pdf]

**Table S3 Summary of the annotation features of the glycosyl- and feruloyl-like coding sequences (CDSs) predicted in the hydrolase-coding DNA fragments from the R library*.**

| Gene name | Strand | Left End | Right End | Length (AA) | Predicted product name | Family | MW (Da) | pI | Organism (best hit) | Expected Value (E). Identities (I). Positives (P) | Top homolog |
|---|---|---|---|---|---|---|---|---|---|---|---|
| *r_01_20* | - | 21563 | 23923 | 786 | beta-Galactosidase | GHF2 | 87991.29 | 5.17 | *Roseburia inulinivorans* | E=0.0; I=441/777 (56%); P=541/777 (69%) | ZP_03752805.1 |
| *r_01_21* | - | 23943 | 26339 | 798 | alpha-Galactosidase | GHF36 | 90107.58 | 5.71 | *Roseburia inulinivorans* | E=0.0; I=482/807 (59%); P=603/807 (74%) | ZP_03752801.1 |
| *r_02-02* | + | 599 | 2191 | 530 | alpha-L-Arabinofuranosidase | GHF51 | 59485.56 | 6.25 | *Prevotella ruminicola* | E=0.0; I=458/530 (86%); P=490/530 (92%) | YP_003573794.1 |
| *r_02-15* | - | 13177 | 14682 | 501 | alpha-Glucosidase | GHF13 | 56950.34 | 5.53 | *Prevotella ruminicola* | E=0.0; I= 398/501 (79%); P= 436/501 (87%) | YP_003574036.1 |
| *r_03-04* | + | 7778 | 8731 | 317 | Beta-Xylosidase | GHF43 | 35572.72 | 5.08 | *Bacteroides intestinalis* | E=2e-102; I=184/316 (58%); P=228/316 (72%) | YP_001297392.1 |
| *r_03-05* | + | 8786 | 10405 | 539 | alpha-L-Arabinofuranosidase | GHF43 | 59988.12 | 7.2 | *Bacteroides xylanisolvens* | E=7e-170; I288/527 (54%=); P=378/527 (71%) | ZP_06769240.1 |
| *r_05-02* | - | 312 | 1949 | 546 | Glycosyl hydrolase family 5 | GHF5 | 61222.36 | 5.31 | Unidentified microorganism | E=0.0; I=328/518 (63%); P=386/518 (74%) | CAJ19140.1 |
| *r_06-02* | + | 1560 | 3632 | 690 | Endo-1,4-beta-xylanase | GHF5 | 78164.3 | 4.87 | *Prevotella ruminicola* | E=5e-142; I=250/403 (62%); P=294/403 (72%) | YP_003575492.1 |
| *r_07-01* | + | 238 | 2718 | 827 | alfa-L-Rhamnosidase | GHF78 | 94254.9 | 4.93 | *Clostridium stercorarium* | E=7e-33; I=352/832 (42%); P=496/832 (59%) | CAB53341.1 |
| *r_07-02* | + | 3436 | 4176 | 260 | beta-Galactosidase | GHF2 | 29543.47 | 8.69 | *Roseburia intestinalis* | E=0.0; I=87/206 (42%); P=117/206 (56%) | CBL10828.1 |
| *r_08-01* | + | 258 | 3027 | 922 | alfa-L-Rhamnosidase | GHF78 | 61801.6 | 5.62 | *Enterococcus casseliflavus* | E=1e-112; I=208/487 (42%); P=300/487 (61%) | ZP_05654693.1 |
| *r_08-02* | + | 3033 | 4069 | 346 | alpha/beta Hydrolase | FAE2 | 40359.5 | 4.95 | *Lysinibacillus sphaericus* | E=7e-42; I=117/306 (38%); P=153/306 (50%) | YP_001698978.1 |
| *r_09-01* | - | 6 | 1148 | 381 | Glycosyl hydrolase family 43 | GHF43 | 44516.1 | 4.93 | *Bryantella formatexigens* | E=3e-115; I=204/383 (53%); P=272/383 (71%) | ZP_05345324.1 |
| *r_09-02* | - | 1157 | 2649 | 496 | Xylosidase/arabinosidase | GHF43 | 54939.62 | 4.96 | *Bacteroides capillosus* | E=4e-120; I=233/502 (46%); P=303/502 (60%) | ZP_02035907.1 |
| *r_09-03* | - | 2691 | 3263 | 189 | beta-Galactosidase | GHF1 | 22244.69 | 4.83 | *Ruminococcus sp.* | E=2e-54; I=108/192 (56%); P=131/192 (68%) | CBL17177.1 |

*The theoretical molecular weight (MW) and isoelectric point (pI) were calculated for the gene product using ExPASy ProtParam online tool.

*Sequence analysis revealed no signal peptide, thereby assuming an intracellular location of the proteins under investigation
