# Supplementary material for: Functional Metagenomics Unveils a Multifunctional Glycosyl Hydrolase from the Family 43 Catalysing the Breakdown of Plant Polymers in the Calf Rumen
Source: PLoS One. 2012 Jun 25;7(6):e38134. doi: 10.1371/journal.pone.0038134 (PMC3382598; doi:10.1371/journal.pone.0038134)
Supplement: Table S4 — Summary of the annotation features of the carbohydrate accessory enzymes identified in the hydrolase-encoding DNA fragments from the R library. (PDF) [file pone.0038134.s012.pdf]

Table S4 Summary of the annotation features of the carbohydrate accessory enzymes identified in the hydrolase-encoding DNA fragments from the R library*.

Gene name	Strand	Left End	Right End	Length (AA)	Gene product name	MW (Da)	pI	Organism (best hit)	Expected Value (E). Identities (I). Positives (P)	Top homolog
r_01_13	+	14147	16546	799	Sugar transporter	89500.04	4.02	*Bacillus mycoides*	E=2e-16; I=111/449 (24%); P=198/449 (44%)	ZP_04160063.1
r_01_22	-	26345	28159	604	Sugar transporter	65605.7	5.14	*Coprococcus comes*	E=1e-86; I=173/302 (57%); P=212/302 (70%)	ZP_06885281.1
r_01_25	+	30423	31670	415	Galactokinase	44143.84	4.93	*Clostridium sp.*	E=7e-139; I=240/405 (59%); P=307/405 (75%)	ZP_02073770.1
r_01_26	+	31682	33166	494	Galactose-1-phosphate uridylyltransferase	55737.38	5.4	*Clostridium sp.*	E=0.0; I=315/498 (63%); P=372/498 (74%)	ZP_02073769.1
r_01_27	+	33237	34520	427	sugar transporter	45007.41	9.13	*Oribacterium sp*	E=3e-117; I=215/423 (50%); P=298/423 (70%)	ZP_06598814.1
r_02-11	+	9203	10285	360	Glycosyltransferase	41162.42	8.23	*Prevotella ruminicola*	E= 3e-169; I= 276/360 (76%); P= 320/360 (88%)	YP_003574041.1
r_02-20	-	17792	20368	858	alpha-Glucan phosphorylase	99019.84	6	*Prevotella ruminicola*	E=0.0; I=766/856 (89%); P=819/856 (95%)	YP_003574031.1
r_02-21	-	20391	22082	563	alpha-Glycosyltransferase	64617.55	5.55	*Prevotella ruminicola*	E=0.0; I=466/576 (80%); P=503/576 (87%)	YP_003574030.1
r_03-01	+	1	3540	1148	Melibiase (CBM)	119432.86	4.48	*Blastopirellula marina*	E=3e-06; I=43/150 (28%); P=77/150 (51%)	ZP_01089974.1
r_03-03	+	6411	7748	445	Sugar transporter	47558.78	9.21	*Bacteriodes xylanisolvens*	E=2e-147; I=267/486 (54%); P=335/486 (68%)	CBK69517.1
r_03-06	+	10683	12005	440	Aldose 1-epimerase	48897.69	9.46	*Opitutus terrae*	E=8e-96; I=172/357 (48%); P=228/357 (63%)	YP_001818271.1

*The theoretical molecular weight (MW) and isoelectric point (pI) were calculated for the gene product using ExPASy ProtParam online tool
