# Supplementary material for: Functional Metagenomics Unveils a Multifunctional Glycosyl Hydrolase from the Family 43 Catalysing the Breakdown of Plant Polymers in the Calf Rumen
Source: PLoS One. 2012 Jun 25;7(6):e38134. doi: 10.1371/journal.pone.0038134 (PMC3382598; doi:10.1371/journal.pone.0038134)
Supplement: Table S5 — Kinetic parameters of the glycosyl and feruloyl hydrolases that were subcloned, expressed, purified and characterised in this study. (PDF) [file pone.0038134.s013.pdf]

Table S5 Kinetic parameters of the glycosyl and feruloyl hydrolases that were subcloned, expressed, purified and characterised in this study.

Enzyme[2]	GHF	Substrate	K_m (mM)	k_{cat} (s^{-1})	k_{cat}/K_m ($s^{-1}M^{-1}$)
R_01-21	GHF36 (α-galactosidase)	*p*NPαGal	0.71 ± 0.10	538.0 ± 45.6	$7.6 \cdot 10^5$
R_02-02	GHF51 (α-arabinofuranosidase)	*p*NPαAf	0.69 ± 0.15	4.1 ± 0.2	$6.1 \cdot 10^3$
		*p*NPαAp	6.40 ± 1.46	118.5 ± 17.2	$1.9 \cdot 10^4$
R_03-04	GHF43 (β-xylosidase)	*p*NPβX	4.12 ± 0.56	127.4 ± 11.7	$3.1 \cdot 10^4$
R_03-05	GHF43 (α-arabinofuranosidase)	*p*NPαAf	4.90 ± 1.06	707.9 ± 15.5	$1.4 \cdot 10^5$
R_07-01	GH78 (α-rhamnosidase)	*p*NPαR	3.58 ± 0.70	$(2.4 \pm 0.2) \cdot 10^3$	$6.8 \cdot 10^5$
R_08-01	GH78 (α-rhamnosidase)	*p*NPαR	3.15 ± 0.30	$(7.2 \pm 0.2) \cdot 10^4$	$2.3 \cdot 10^7$
R_08-02	FAE (feruloyl esterase)	*p*NPA	2.73 ± 0.91	7.0 ± 0.3	$2.6 \cdot 10^3$
		*p*NPP	2.21 ± 0.44	54.6 ± 7.0	$2.5 \cdot 10^4$
		MF	3.21 ± 0.39	28.7 ± 2.1	$8.9 \cdot 10^3$
		MpC	3.50 ± 2.25	30.7 ± 5.1	$8.7 \cdot 10^3$

Parameters were determined at the optimal pH and temperature, namely pH 5.5 and 45°C for R_01-21, pH 5.5 and 45°C for R_02-02, pH 6.0 and 35°C for R_02-15, pH 5.5 and 50°C for R_03-04 and R_03-05, pH 8.5 and 55°C for R_07-01, pH 5.5 and 50°C for R_08-01, pH 8.0 and 50°C for R_08-02, pH 5.5 and 45°C for R_09-02. *K*m, *k*cat and *K*m values were obtained as described as follows: [E]o= 0-12 nM, [substrate] ranging from 0 to 100 mM.

* Truncated genes that were not successfully cloned and purified as follows: R_01-20 (GHF2 β-galactosidase), R_03-01 (CMB_51), R_05-01 (GHF5 endo-β-1,4-glucanase), R_06-02 (GHF5 endo-β-1,4-glucanase), R_07-02 (GHF2), R_09-01 (GHF43) and R_09-03 (GHF1 β-galactosidase).
