# Supplementary material for: Functional Metagenomics Unveils a Multifunctional Glycosyl Hydrolase from the Family 43 Catalysing the Breakdown of Plant Polymers in the Calf Rumen
Source: PLoS One. 2012 Jun 25;7(6):e38134. doi: 10.1371/journal.pone.0038134 (PMC3382598; doi:10.1371/journal.pone.0038134)
Supplement: Table S6 — Vectors (A) and oligonucleotides (B) used in this work. (PDF) [file pone.0038134.s014.pdf]

Table S6 Vectors (A) and oligonucleotides (B) used in this work.

(A) Vectors

**Fosmid/plasmid**	**Description**
r_01	pCCFOS vector with a 34609 bp of rumen derived DNA fragment containing the hydrolases genes *r_01-20* (2349 bp) and *r_01-21* (2394 bp)
r_02	pCCFOS vector with a 26383 bp of rumen derived DNA fragment containing the hydrolases genes *r_02-02* (1060 bp) and *r_02-15* (1503 bp)
r_03	pCCFOS vector with a 26679 bp of rumen derived DNA fragment containing the hydrolases genes *r_03-04* (951 bp) and *r_03-05* (1317 bp)
r_05	pBK-CMV vector containing the 4699 bp of rumen derived metagenomic DNA harbouring the 1641 bp coding region of *r_05-01*
r_06	pBK-CMV vector containing the 5866 bp of rumen derived metagenomic DNA harbouring the 2073 bp coding region of *r_06-02*
r_07	pUC19 vector containing the 4215 bp of rumen metagenomic DNA harbouring the coding region of *r_07-01* (2484 bp) and *r_07-02* (831 bp)
r_09	pUC19 vector containing the 3305 bp of metagenomic DNA harbouring the 1491 bp coding region of *r_09-02*
pGEM-*r_01-21*	pGEM-T Easy vector containing the 2397 bp gene coding R_01-21 hydrolase
pGEM-*r_02-02*	pGEM-T Easy vector containing the 1593 bp gene coding R_02-02 hydrolase
pGEM-*r_02-15*	pGEM-T Easy vector containing the 1506 bp gene coding R_02-15 hydrolase
pGEM-*r_03-4*	pGEM-T Easy vector containing the 951 bp gene coding R_03-4 hydrolase
pGEM-*r_03-5*	pGEM-T Easy vector containing the 1317 bp gene coding R_03-5 hydrolase
pGEM-*r_07-01*	pGEM-T Easy vector containing the 2484 bp gene coding R_07-01 hydrolase
pGEM-*r_08-01*	pGEM-T Easy vector containing the 1668 bp gene coding R_08-01 hydrolase
pGEM-*r_08-02*	pGEM-T Easy vector containing the 1101 bp gene coding R_08-02 hydrolase
pGEM-*r_09-02*	pGEM-T Easy vector containing the 1491 bp gene coding R_09-02 hydrolase
pET-41Ek/LIC-*r_01-21*	pET-41Ek/LIC vector containing the 2397 bp gene coding R_01-21 hydrolase
pET-41Ek/LIC-*r_02-02*	pET-41Ek/LIC vector containing the 1593 bp gene coding R_02-02 hydrolase
pET-41Ek/LIC-*r_02-15*	pET-41Ek/LIC vector containing the 1506 bp gene coding R_02-15 hydrolase
pET-41Ek/LIC-*r_03-4*	pET-41Ek/LIC vector containing the 951 bp gene coding R_03-4 hydrolase
pET-41Ek/LIC-*r_03-5*	pET-41Ek/LIC vector containing the 1317 bp gene coding R_03-5 hydrolase
pET-41Ek/LIC-*r_07-01*	pET-41Ek/LIC vector containing the 2484 bp gene coding R_07-01 hydrolase
pET-41Ek/LIC-*r_08-01*	pET-41Ek/LIC vector containing the 1668 bp gene coding R_08-01 hydrolase
pET-41Ek/LIC-*r_08-02*	pET-41Ek/LIC vector containing the 1101 bp gene coding R_08-02 hydrolase
pET-41Ek/LIC-*r_09-02*	pET-41Ek/LIC vector containing the 1491 bp gene coding R_09-02 hydrolase

Table S6 (B) Primers

**Fosmid/plasmid**	**Oligonucleotides**
*r_01-21*_Fwd	5’-gacgacgacaagATGATCACTGTTAAGGGGGATAAGACCC-3’
*r_01-21*_Rev	5’-gaggagaagcccggTTACTTATCGATCTTCTCTATCGTGTATAACC-3’
*r_01-20*_Fwd	5’-gacgacgacaagATGAGGATCGACATCAATAACGACTGGC-3’
*r_01-20*_Rev	5’-gaggagaagcccggTTACGCCAGACGCACGGTGAAGTCCGCC-3’
*r_02-15*_Fwd	5’-gacgacgacaagATGAAGAAACTATTCATTACCGCAACG -3’
*r_02-15*_Rev	5’-gaggagaagcccggTTATTTGATGTTTAATACGATTATCGATTTTGC -3’
*r_02-02*_Fwd	5’-gacgacgacaagATGGGCGTGAGTCATATCTGGTACACGGG -3’
*r_02-02*_Rev	5’-gaggagaagcccggTTATGCTTTGAATTTCAGAATTAACGCGCC -3’
*r_03-04*_Fwd	5’-gacgacgacaagATGAACGGTGCCGGAACGCTCAAACTC-3’
*r_03-04*_Rev	5’-gaggagaagcccggTTATCGCACGATCACAACGAACGCGTCGCC-3’
*r_03-05*_Fwd	5’-gacgacgacaagATGAAAACAGGGATGCTACTTTCGCTCAC-3’
*r_03-05*_Rev	5’-gaggagaagcccggTTACCGCAGAGAACCCACGTGCGGTTGCGG-3’
*r_07-01*_Fwd	5’-gacgacgacaagATGAAGCTGAACGATCTGCGCGTCTGC-3’
*r_07-01*_Rev	5’-gaggagaagcccggTTATGCTTCCTCCTCCCAGTGATAGCTGCC-3’
*r_08-01*_Fwd	5’-gacgacgacaagATGAAACTTGAAGCGCTGAAGATCAACGG-3’
*r_08-01*_Rev	5’-gaggagaagcccggTTAAAGCTGTAGTGCCGCCTGGTGTCCATCC-3’
*r_08-02*_Fwd	5’-gacgacgacaagATGCGACTGGACAGCAAGCTGCCCC-3’
*r_08-02*_Rev	5’-gaggagaagcccggTTATCCGTTCAGTTTTCCGTCAAATACGCCG-3’
*r_09-02*_Fwd	5’-gacgacgacaagATGGAACAGGTTTTCAATCCCTATCTGCC-3’
*r_09-02*_Rev	5’-gaggagaagcccggTTACTCTGCAAAAAAACAGATTCTTTTCAGCTCC-3’
