# Supplementary material for: Functional Metagenomics Unveils a Multifunctional Glycosyl Hydrolase from the Family 43 Catalysing the Breakdown of Plant Polymers in the Calf Rumen
Source: PLoS One. 2012 Jun 25;7(6):e38134. doi: 10.1371/journal.pone.0038134 (PMC3382598; doi:10.1371/journal.pone.0038134)
Supplement: Table S7 — Biochemical information of GHF43 enzymes described in Figure 2 . Data are based on bibliographic records that are specifically cited. (PDF) [file pone.0038134.s015.pdf]

Table S7 Biochemical information of GHF43 enzymes described in Figure 2. Data are based on bibliographic records that are specifically cited.

Uniprot/GenBank code	Source	Substrates tested	Reference
P07129<br>(β-Xylosidase)	*Bacillus pumilus*	Active towards *p*NPβXyl (V_m 1.23 ± 0.14 µkat/mg; K_m 3.9 ± 0.59 mM) and xylobiose (V_m 0.14 ± 0.011 µkat/mg; K_m 8.99 ± 1.19 mM)<br>No activity towards xylan (< 0.8 nkat/mg)	Xu WZ, Shima Y, Negoro S, Urabe I. (1991). Sequence and properties of beta-xylosidase from *Bacillus pumilus* IPO. Contradiction of the previous nucleotide sequence. *Eur. J Biochem* **202:** 1197-1203
Q9Z477<br>(β-Xylosidase)	*Bacillus pumilus*	Active towards *p*NPβXyl (no activity value indicated)	La Grange DC, Pretorius IS, van Zyl WH. (1997). Cloning of the Bacillus pumilus beta-xylosidase gene (xynB) and its expression in *Saccharomyces cerevisiae. Appl. Microbiol. Biotechnol.* **47:** 262-266
P94489<br>(β-Xylosidase)	*Bacillus subtilis*	Active towards *p*NPβXyl (43.5 units per 1.4x10^9 cellsml^{-1}min^{-1}), xylan (0.27 units per 1.4x10^9 cellsml^{-1}min^{-1})	Roncero MI. (1983). Genes controlling xylan utilization by *Bacillus subtilis*. *J Bacteriol.* **156:** 257-263
O52575<br>(β-Xylosidase/α-Arabinosidase)<br>(Xyl > Ara)	*Selenomonas ruminantum*	Active towards *p*NPαAraf (1.8 U/mg), *p*NPβXyl (15.7 U/mg) and xylooligosacharides (not given)<br>No activity towards xylan	Whitehead TR, Cotta MA. (2001). Identification of a broad-specificity xylosidase/arabinosidase important for xylooligosaccharide fermentation by the ruminal anaerobe *Selenomonas ruminantium* GA192. *Curr. Microbiol.* **43:** 293-298
Q09LX0<br>(β-Xylosidase/α-Arabinosidase)<br>(Xyl > Ara)	*Geobacillus stereatermphilus*	Active towards *p*NPβXyl (k_{cat} 57 s^{-1}; K_m 17 mM) and *p*NPαAraf (k_{cat} 2.7 s^{-1}; K_m 6 mM)<br>No activity towards *p*NPαGal, *p*NPαArap, *p*NPαGlu, *p*NPαFuc, *p*NPαRhamn and *p*NPβMan (k_{cat} < 0.029 s^{-1})	Shallom D, Leon M, Bravman T, Ben-David A, Zaide G, et al. (2005). Biochemical characterization and identification of the catalytic residues of a family 43 beta-D-xylosidase from *Geobacillus stearothermophilus* T-6. *Biochemistry* **44:** 387-397
Q9K6P5<br>(β-Xylosidase/α-Arabinosidase)<br>(Xyl > Ara)	*Bacillus halodurans*	Active towards *p*NPβXyl (k_{cat} 12.09s^{-1}; K_m 4.40 mM) and *p*NPαAraf (k_{cat} 2.11s^{-1}; K_m 13.84 mM)	Smaali I, Remond C, O'Donohue MJ. (2006). Expression in Escherichia coli and characterization of beta-xylosidases GH39 and GH-43 from *Bacillus halodurans* C-125. *Appl. Microbiol. Biotechnol.* **73:** 582-590
A9Z9D1<br>(β-Xylosidase)	*Vibrio* sp.	Active towards *p*NPβXyl (V_{max} 1.82 µmolmin^{-1}mg^{-1}; K_m 0.244 mM) and xylo-oligosacharides (1,3) > (1,4) (not given)<br>No activity towards *p*NPαXyl, *p*NPαGal, *p*NP*p*Araf, *p*NPαArap, *p*NPαGlu, *p*NPβGlu , *p*NPαFuc, *p*NPαMan, *p*NPβMan, *p*NPβCel, *p*NPβglucuronide and xylan	Umemoto Y, Onishi R, Araki T. (2008). Cloning of a novel gene encoding beta-1,3-xylosidase from a marine bacterium, *Vibrio* sp. strain XY-214, and characterization of the gene product. *Appl. Environ. Microbiol.* **74:** 305-308
P45982<br>(α-Arabinosidase/β-Xylosidase/α-Glucosidase)	*Butyvibrio fibrisolvens*	Active towards *p*NPβXyl (8.9 nmolmin^{-1}mg^{-1}), *p*NPαAraf (15.5 nmolmin^{-1}mg^{-1}), *p*NPαArap (0.14 nmolmin^{-1}mg^{-1}), *p*NPαGal (0.17 nmolmin^{-}	Utt EA, Eddy CK, Keshav KF, Ingram LO. (1991). Sequencing and expression of the *Butyrivibrio fibrisolvens* xylB gene encoding a novel bifunctional protein with beta-D-xylosidase and alpha-L-

		^{1}mg^{-1}), *p*NPαGlu (3.25 nmolmin^{-1}mg^{-1}), *p*NPαMan (0.15 nmolmin^{-1}mg^{-1}), *p*NPβMan (0.10 nmolmin^{-1}mg^{-1}), *p*NPαFuc (0.19 nmolmin^{-1}mg^{-1}), *p*NPβDFuc (0.22 nmolmin^{-1}mg^{-1}), *p*NPβLFuc (0.42 nmolmin^{-1}mg^{-1}), *p*NPαRhamn (0.28 nmolmin^{-1}mg^{-1})	arabinofuranosidase activities. *Appl. Environ. Microbiol.* **57:** 1227-1234
A055D8 (α-Arabinosidase/β-Xylosidase) (Ara > Xyl)	Uncultured bacterium	Active towards *p*NPαAraf (k_{cat} 0.684s^{-1}; K_m 0.25 mM), *p*NPβXyl k_{cat} 0.132s^{-1}; K_m 0.96mM) and arabinan (not given)<br>No activity towards *p*NPαArap, *p*NPβGlu, *p*NPβDFuc, *p*NPβGal, *p*NPβMan, xylobiose and xylan	Wagschal K, Franqui-Espiet D, Lee CC, Kibblewhite-Accinelli RE, Robertson GH, Wong DW. (2007). Genetic and biochemical characterization of an α-L-arabinofuranosidase isolated from a compost starter mixture. *Enzyme and Microbial Technology* **40:** 747-753
B8QP77 (β-Xylosidase/α-Arabinosidase) (Xyl > Ara)	Uncultured bacterium	Active towards *p*NPαAraf (k_{cat} 0.120 s^{-1}; K_m 0.072mM), *p*NPβXyl (k_{cat} 1.34 s^{-1}; K_m 1.53 mM), xylobiose (k_{cat} 0.047 s-1; K_m 15.9mM), xylotriose (k_{cat} 0.044 s^{-1}; K_m 10.4 mM), xylotetraose (k_{cat} 0.019 s^{-1}; K_m 7.5 mM), arabinan, arabinobiose, arabinotriose and arabinoxylan<br>No activity towards *p*NPαXyl, *p*NPαArap, *p*NPβArap, *p*NPαGlu, *p*NPβGlu, *p*NPαLFuc, *p*NPβDFuc, *p*NPαGal, *p*NPβGal, *p*NPαMan and *p*NPβMan	Wagschal K, Heng C, Lee CC, Wong DW. (2009). Biochemical characterization of a novel dual-function arabinofuranosidase/xylosidase isolated from a compost starter mixture. *Appl. Microbiol. Biotechnol.* **81:** 855-863
Q76EC8 (β-Xylosidase)	*Clostridium stercorarium*	Active towards *p*NPβXyl (10 U/mg; V_{max} 15 μmolmin^{-1}mg^{-1}; K_m 6.3 mM) and xylan (0.05-0.1 U/mg)<br>No activity towards *p*NPαXyl, *p*NPαArap and *p*NPαLAraf	Suryani, Kimura T, Sakka K, Ohmiya K. (2004). Sequencing and expression of the gene encoding the *Clostridium stercorarium* beta-xylosidase Xyl43B in *Escherichia coli*. *Biosci. Biotechnol. Biochem* **68:** 609-614
A1A049 (α-Arabinosidase)	*Bifidobacterium adolescentis*	Active towards arabinoxylanoligosacharides (arabinose release) (90.4 U/mg) and *p*NPαAraf (0.095 U/mg)<br>No activity towards xylan and arabinan	van den Broek LA, Lloyd RM, Beldman G, Verdoes JC, McCleary BV, Voragen AG. (2005). Cloning and characterization of arabinoxylan arabinofuranohydrolase-D3 (AXHd3) from *Bifidobacterium adolescentis* DSM20083. *Appl. Microbiol. Biotechnol.* **67:** 641-647
P45796 (α-Arabinosidase)	*Paenibacillus polymixa*	Active towards wheat flour xylan, oat spelt xylan (arabinose release) (91-135 unitsmin^{-1}mg^{-1}) and *p*NPαAraf (0.98 unitsmin^{-1}mg^{-1})<br>No activity towards *p*NPβXyl, birchwood xylan, arabinan, arabinogalactan, xylo-oligosacharides and arabino-oligosacharides	Morales P, Sendra JM, Perez-Gonzalez JA. (1995). Purification and characterization of an arabinofuranosidase from Bacillus polymyxa expressed in *Bacillus subtilis*. *Appl. Microbiol. Biotechnol.* **44:** 112-117
Q45071 (α-Arabinosidase)	*Bacillus subtilis*	Active towards *p*NPαAraf (74 mU/mg), arabinooligosacharides (3.2 mU/mg), arabinoxylan (1.8 mU/mg) (arabinose release), arabinan (< 0.01 mU/mg) and *p*NPβXyl (< 0.01	Bourgois TM, Van C, V, Van CS, Courtin CM, Delcour JA, Robben J, Volckaert G. (2007). Recombinant expression and characterization of XynD from *Bacillus subtilis* subsp. *subtilis* ATCC 6051: a GH 43 arabinoxylan arabinofuranohydrolase. *Appl. Microbiol. Biotechnol.*

		mU/mg)	**75:** 1309-1317
B3PD60<br>(α-Arabinosidase)	*Cellvibrio japonicus*	Active towards sugar beet arabinan (arabinose release) and *p*NPαLAraf<br>No activity towards linear arabinan, xylan, pectin, β-glucan and *p*NPβDXyl	Cartmell A, McKee LS, Pena MJ, Larsbrink J, Brumer H, et al. (2011). The structure and function of an arabinan-specific alpha-1,2-arabinofuranosidase identified from screening the activities of bacterial GH43 glycoside hydrolases. *J Biol. Chem.* **286:** 15483-15495
ADV16404<br>(α-Arabinosidase/β-Xylosidase)	*Paenibacillus woosongiensis*	Active towards *p*NPβXyl (660 mU/mg; V_{max} 1.4 µmolmin^{-1}mg^{-1}; K_m 1.1 mM), *p*NPαAraf (mU/mg; Vmax 3.1 µmolmin^{-1}mg^{-1}; K_m 8.5 mM), *p*NPαArap (0.06 mU/mg), *p*NPβGal (0.65 mU/mg) and xylan (<0.03 mU/mg)<br>No activity towards *p*NPβGlu, *p*NPβGal and *p*NPβMan, *p*NPαGal, , *p*NPαGlu	Kim YA, Yoon KH. (2010). Characterization of a *Paenibacillus woosongensis* beta-xylosidase/alpha-arabinofuranosidase produced by recombinant *Escherichia coli*. *J Microbiol. Biotechnol.* **20:** 1711-1716
P48790<br>(α-Arabinosidase/β-Xylosidase)	*Clostridium stercorarium*	Active towards *p*NPβXyl (V_{max} 5.9 nmolmin^{-1}µg^{-1}; K_m 2.5 mM), *p*NPαAraf (Vmax 16.7 nmolmin^{-1}µg^{-1}; K_m 17.6 mM) and xylobiose (not given)<br>No activity towards *p*NPβGlu, *p*NPβGlu and *p*NPβMan	Sakka K, Yoshikawa K, Kojima Y, Karita S, Ohmiya K, Shimada K. (1993). Nucleotide sequence of the *Clostridium stercorarium* xylA gene encoding a bifunctional protein with beta-D-xylosidase and alpha-L-arabinofuranosidase activities, and properties of the translated product. *Biosci. Biotechnol. Biochem* **57:** 268-272
D8VNC7<br>(β-Galactosidase)	Uncultured bacterium	Active towards *p*NPβGal (k_{cat} 1780 s^{-1}; K_m 0.19 mM), *p*NPαArap (k_{cat} 1800 s^{-1}; K_m 13.1 mM), *p*NPαAraf (k_{cat} 1950 s^{-1}; K_m 13.1 mM) and *p*NPβXyl (k_{cat} 2800 s^{-1}; K_m 22.8 mM)	Beloqui A, Nechitaylo TY, Lopez-Cortes N, Ghazi A, Guazzaroni ME, et al. (2010). Diversity of glycosyl hydrolases from cellulose-depleting communities enriched from casts of two earthworm species. *Appl. Environ. Microbiol.* **76:** 5934-5946
P48791<br>(β-Xylosidase/α-Arabinosidase)	*Prevotella bryantii*	Active towards *p*NPβXyl (101 nmolmin^{-1}mg^{-1}), *p*NPαAra (89 nmolmin^{-1}mg^{-1}), xylan (22-540 nmolmin^{-1}mg^{-1}), xylobiose (not given) and xylopentaose (not given)	Gasparic A, Martin J, Daniel AS, Flint HJ. (1995). A xylan hydrolase gene cluster in *Prevotella ruminicola* B(1)4: sequence relationships, synergistic interactions, and oxygen sensitivity of a novel enzyme with exoxylanase and beta-(1,4)-xylosidase activities. *Appl. Environ. Microbiol.* **61:** 2958-2964
B3FRL6<br>(α-Arabinosidase)	*Bacillus subtilis*	Active towards linear α-1,5-L-arabinan (73 U/mg), branched sugar beet arabinan (51 U/mg) and pectin (10 U/mg)<br>No activity towards larchwood arabinogalactan, arabinoxylan and *p*NPαAraf	Inacio JM, de Sa-Nogueira I. (2008). Characterization of abn2 (yxiA), encoding a *Bacillus subtilis* GH43 arabinanase, Abn2, and its role in arabino-polysaccharide degradation. *J Bacteriol.* **190:** 4272-4280
D2XML7<br>(α-Arabinosidase)	Uncultured bacterium	Active towards CM-arabinan, debranched arabinan, linear arabinan, sugar beet arabinan and arabinooligosacharides (DP>5)<br>No activity towards arabinoxylan and pNPαAraf	Wong DW, Chan VJ, McCormack AA. (2009). Functional cloning and expression of a novel endo-alpha-1,5-L-arabinanase from a metagenomic library. *Protein Pept. Lett.* **16:** 1435-1441
BAC68753<br>(α-Arabinosidase)	*Sreptomyces avertimiles*	Active towards *p*NPαAraf, debranched arabinan and α(1,5)arabinofuranooligosaccharides (values not given)	Ichinose H, Yoshida M, Fujimoto Z, Kaneko S. (2008). Characterization of a modular enzyme of exo-1,5-alpha-L-arabinofuranosidase and arabinan binding module from *Streptomyces*

Eliminado: No activity tested towards *p*NPβXyl

Eliminado: No activity tested towards *p*NPβXyl

		No activity towards *p*NPαArap, *p*NPβXyl, *p*NPβGal, xylan, arabinoxylan and arabinan	*avermitilis* NBRC14893. *Appl. Microbiol. Biotechnol.* **80:** 399-408<br>Fujimoto Z, Ichinose H, Maehara T, Honda M, Kitaoka M, Kaneko S. (2010). Crystal structure of an Exo-1,5-{alpha}-L-arabinofuranosidase from *Streptomyces avermitilis* provides insights into the mechanism of substrate discrimination between exo- and endo-type enzymes in glycoside hydrolase family 43. *J Biol. Chem.* **285:** 34134-34143
P82594<br>(α-Arabinosidase)	*Streptomyces chartreusis*	Active towards *p*NPαAraf (not given), α(1,5)linked arabinan (4.3-35.7% activity), arabinoxylan (2.6-5.2% activity) and arabinogalactan (2.2-2.4% activity) (arabinose release)<br>No activity towards *p*NPαArap , *p*NPβXyl , *p*NPβGal , gum Arabic and arabinoxylooligosacharides	Matsuo N, Kaneko S, Kuno A, Kobayashi H, Kusakabe I. (2000). Purification, characterization and gene cloning of two alpha-L-arabinofuranosidases from *Streptomyces chartreusis* GS901. *Biochem J* **346:** 9-15
P94522<br>(α-Arabinosidase)	*Bacillus subtilis*	Active towards linear α(1,5)linked arabinan and sugar beet arabinan (arabinose release)<br>No activity towards arabinogalactan, arabinoxylan, pNPαAraf	Leal TF, de Sa-Nogueira I. (2004). Purification, characterization and functional analysis of an endo-arabinanase (AbnA) from *Bacillus subtilis*. *FEMS Microbiol. Lett.* **241:** 41-48
B3EYM8<br>(α-Arabinosidase)	*Geobacillus stereatermophilus*	Active towards debranched arabinan, arabino-oligosacharides (DP 2-8) (data not given)<br>No activity towards *p*NPαAraf, *p*NPαArap and *p*NPβXyl	Alhassid A, Ben-David A, Tabachnikov O, Libster D, Naveh E, et al. (2009). Crystal structure of an inverting GH 43 1,5-alpha-L-arabinanase from *Geobacillus stearothermophilus* complexed with its substrate. *Biochem J* **422:** 73-82
Q93HT9<br>(α-Arabinosidase)	*Geobacillus thermodenitrificans*	Active towards debranched arabinan (445 U/mg)	Takao M, Yamaguchi A, Yoshikawa K, Terashita T, Sakai T. (2002). Molecular cloning of the gene encoding thermostable endo-1,5-alpha-L-arabinase of *Bacillus thermodenitrificans* TS-3 and its expression in Bacillus subtilis. *Biosci. Biotechnol. Biochem* **66:** 430-433
B3PKP8<br>(α-Arabinosidase)	*Cellvibrio japonicus*	Active towards linear arabinan (k_{cat} 109 s^{-1}; K_m 3.75 mg/ml), arabino-oligosacharides (arabinotriose release; from 200-2174 units (relative activity) from arabinotetraose to arabino-octaose)<br>No activity towards branched sugar beet arabinan, cellulose, xylan, galactan, mannan, *p*NPαAraf, *p*NPαArap, *p*NPβXyl and 2,4-dinitrophenyl xylobioside	McKie VA, Black GW, Millward-Sadler SJ, Hazlewood GP, Laurie JI, Gilbert HJ. (1997). Arabinanase A from *Pseudomonas fluorescens* subsp. *cellulosa* exhibits both an endo- and an exo- mode of action. *Biochem J* **323:** 547-555
D2XML8<br>(α-Arabinosidase)	Uncultured bacterium	Active towards linear CM-arabinan (43.2 μmolmin^{-1}mg^{-1}; V_{max} 0.86 mgml^{-1}min^{-1}; K_m 6.2 mg/ml), debranched arabinan (34.9 μmolmin^{-1}mg^{-1}), linear arabinan (23.2 μmolmin^{-1}mg^{-1}) and arabino-oligosacharides (not given)(arabinose release)	Wong DW, Chan VJ, Batt SB. (2008). Cloning and characterization of a novel exo-alpha-1,5-L-arabinanase gene and the enzyme. *Appl. Microbiol. Biotechnol.* **79:** 941-949

		No activity towards sugar beet arabinan, arabinoxylan and *p*NPαAraf	

Abbreviations as follows: *p*NPβXyl: *p*-nitrophenyl-β-D-xylopyranoside; *p*NPαAraf: *p*-nitrophenyl-α-L-arabinofuranoside; *p*NPαGal: *p*-nitrophenyl-α-D-galactoside; *p*NPβGal: *p*-nitrophenyl-β-D-galactoside; *p*NPαArap: *p*-nitrophenyl-α-L-arabinopyranoside; *p*NPαGlu: *p*-nitrophenyl-α-D-glucoside; *p*NPαFuc: *p*-nitrophenyl-α-L-fucoside; *p*NPαRhamn: *p*-nitrophenyl-α-L-rhamnoside; *p*NPβMan: *p*-nitrophenyl-β-D-mannoside; *p*NPαXyl: *p*-nitrophenyl-α-D- xylopyranoside; *p*NPβGlu: *p*-nitrophenyl-β-D- glucoside; *p*NPαMan: *p*-nitrophenyl-α-D-mannoside; *p*NPβCel: *p*-nitrophenyl-β-D-cellobioside; *p*NPβglucuronide: *p*-nitrophenyl-β-D-glucuronide; *p*NPβDFuc: *p*-nitrophenyl-β-D-fucoside; *p*NPβLFuc: *p*-nitrophenyl-β-L-fucoside; CM-arabinan: carboxymethyl arabinan.

.
