# Supplementary material for: Functional Metagenomics Unveils a Multifunctional Glycosyl Hydrolase from the Family 43 Catalysing the Breakdown of Plant Polymers in the Calf Rumen
Source: PLoS One. 2012 Jun 25;7(6):e38134. doi: 10.1371/journal.pone.0038134 (PMC3382598; doi:10.1371/journal.pone.0038134)
Supplement: Methods S1 — Complete description of materials and methods and cloning, expression and purification of the plant polymeric-substance hydrolases. (DOC) [file pone.0038134.s016.doc]

**SUPPORTING METHODS**

**Materials and strains**

Chemicals, biochemicals and solvents were purchased from Sigma-Fluka-Aldrich Co. (St. Louis, MO, USA) and were of p.a. (pro-analysis) quality. The oligonucleotides used for DNA amplification, mutagenesis and sequencing were synthesised by Sigma Genosys Ltd. (Pampisford, Cambs, UK). The restriction and modifying enzymes were from New England Biolabs (Beverly, MA, USA). The Ni-NTA His⋅Bind chromatographic media were from QIAGEN (Hilden, Germany). The *E. coli* EPI300-T1^R^ strain (Epicentre Biotechnologies; Madison, WI, USA) used for the fosmid library construction and screening, XL10 Gold (Invitrogen (Carlsbad, CA, USA) for the site-directed mutagenesis and *E. coli* GigaSingles for the cloning and BL21(DE3)pLysS for the expression using the pET-30 Ek/LIC vector (Novagen, Darmstadt, Germany) were cultured and maintained according to the recommendations of the suppliers.

The following substrates were used for the activity testing and screening: *p-*nitrophenyl (*p*NP) α-L-arabinofuranoside (*p*NPαAf); *p*NP-α- and *p*NP-β-galactopyranoside (*p*NPαGal and *p*NPβGal); *p*NP-α- and *p*NP-β-xylopyranoside (*p*NPαX and *p*NPβX); *p*NP-β-D-glucopyranoside (*p*NPβG); *p*NP-β-D-cellobioside (*p*NPβC); *p*NP-α-L-rhamnopyranoside (*p*NPαR) and carboxymethyl cellulose (CMC); *p*NP-α- and *p*NP-β-arabinopyranoside (*p*NPαAp and *p*NPβAp); *p*NP-acetate (*p*NPA); *p*NP-propionate (*p*NPP); *p*NP-butyrate (*p*NPB); methyl ferulate (MF); methyl sinapinate (MS); methyl-*p*-coumarate (MpC) and methyl cinnamate (MC). All of the *p*NP derivatives were purchased from Sigma-Fluka-Aldrich Co. (St. Louis, MO, USA). MF, MS, MC and MpC were from Apin Chemicals (Oxon, UK). The xylo-oligosaccharides (ranging from xylobiose to xylohexaose), arabino-oligosaccharides (ranging from arabinobiose to arabinooctaose), xyloglucan, a higher-degree polymerised xyloglucan oligocaccharide, and kits for D-xylose and L-arabinose assays were all provided by Megazyme International (Bray, Ireland).

**Cloning, expression and purification of the plant polymeric-substance hydrolases**

The gene cloning was performed by PCR using PfuTurbo DNA polymerase and custom oligonucleotide primers. To amplify the hydrolase genes, the corresponding fosmid or plasmid was used as the template; the vectors and the pairs of primers are described in **Tables S7** and **S8**. The PCR conditions were as follows: 95 °C for 120 s, followed by 30 cycles of 95 °C for 45 s, 50 °C for 60 s and 72 °C for 120 s, with a final annealing at 72 °C for 500 s. The PCR products were purified from agarose gels after electrophoresis using the QiaExII Gel Extraction Kit (Qiagen, Hilden, Germany) and cloned into pET-41-Ek/LIC (Novagen) according to the manufacturer’s instructions. The plasmids were subsequently isolated and introduced into the non-expressing *E. coli* GigaSingles host and also into *E. coli* BL21 (DE3)pLysS for protein expression; the clones were selected on Luria-Bertani (LB) agar supplemented with kanamycin (30 μg/mL). For the enzyme expression and purification, randomly selected clones were grown overnight at 37 ºC with shaking at 200 rpm in 100 mL of LB medium containing kanamycin. Afterwards, 25 mL of this culture was used to inoculate 1 L of LB medium, which was then incubated for 4 hours to an OD_600nm_ of ~0.6. Protein expression was induced by 1 mM isopropyl-β-D-galactopyranoside (IPTG) followed by incubation for 5-6 h. The cells were harvested by centrifugation at 5000 × *g* for 15 min to yield 2-3 g/L of pellet (wet weight). The cell pellet was frozen at -80 ºC overnight, thawed and resuspended in 10 mL/g lysis buffer (20 mM HEPES [pH 7.0], 0.3 M NaCl, 5 mM imidazole and 2 μg/ml DNase). Lysozyme (1 mg/mL) was added and incubated for 1 h on ice with occasional mixing. The cell suspension was then sonicated for a total of 1.2 min and centrifuged at 15 000 × *g* for 15 min; the supernatant was retained. The His_6_-tagged enzymes were purified at 25 °C after binding to a Ni-NTA His⋅Bind resin. The columns were pre-washed with 20 mM HEPES (pH 7.0), 0.3 M NaCl and 5 mM imidazole, and the enzymes were eluted with 20 mM HEPES (pH 7.0), 0.3 M NaCl and 250 mM imidazole. The monitoring of the enzyme elution was performed by SDS-PAGE and/or activity measurements, using standard assays. The purity was assessed as >95% using SDS-PAGE, which was performed with 12% (v/v) polyacrylamide gels, as described by Laemmli [43], using a Bio-Rad Mini Protean system. The protein concentrations were determined according to Bradford [44] using bovine serum albumin as the standard.

The expression of R_09-02 was performed at three different temperatures, as described below. The cells were pre-grown overnight at 37 ºC with shaking at 200 rpm in 100 mL of LB medium containing kanamycin (30 μg/mL), and 4 ml of this culture was used as the inoculum for 200 mL of medium (OD_600nm_ of 0.05), and the cells were grown for 2 h to an OD_600nm_ of ~0.6. The cultures were then grown at three different temperatures (37, 28 and 22 ºC) after the addition of 0.5 mM IPTG to induce protein expression. Samples (1 mL) were collected at different time points to determine the protein concentration (Bradford, 1976) and expression [43], and the activity levels using *p*NPβX as the substrate (see above) were analysed. The optimal conditions were defined at 28 ºC for 7 h of induction, and the cells grown under these conditions were used for the protein extraction and purification, as described above.
