# Supplementary material for: Functional Metagenomics Unveils a Multifunctional Glycosyl Hydrolase from the Family 43 Catalysing the Breakdown of Plant Polymers in the Calf Rumen
Source: PLoS One. 2012 Jun 25;7(6):e38134. doi: 10.1371/journal.pone.0038134 (PMC3382598; doi:10.1371/journal.pone.0038134)
Supplement: Text S1 — Complete description of rumen degradative enzymes (phylogeny and biochemistry), analysis of the DNA fragments using genome linguistics and 3-D modelling analysis of microbial hydrolases from the R library. (DOC) [file pone.0038134.s017.doc]

**SUPPORTING TEXT**

**Rumen degradative enzymes: phylogeny and biochemistry**

*Analysis of the GHs encoded by the sequences in the r_01 fosmid* **−** The DNA insert sequence of the positive clone, r_01, was 34 609 bp in length, with a G+C content of 53.78%. This sequence harboured 27 predicted protein-encoding ORFs, one of which encoded a putative GHF2 (R_01-20) and another encoded a GHF36 (R_01-21). Overall, 13 out of 27 deduced ORFs were significantly similar (30%-63% amino acid [AA] sequence identity) to known proteins from members of *Firmicutes,* such as *Coprococcus*, *Ruminococcus*, *Clostridium* and *Roseburia*. The remaining 12 hypothetical proteins had no deduced function and were very similar to sequences from uncultivated *Clostridia* (**Table S2A**).

The predicted R_01-20 (at nucleotide positions 21 563-23 923) and R_01-21 (at positions 23 943-26 339) proteins have predicted molecular masses (Mm) of 87 991 and 90 107 Da and predicted isoelectric points (*p*I) of 5.17 and 5.71, respectively. These putative proteins exhibited 56 and 59% AA sequence identity to β-galactosidase [ZP_03752805.1](http://www.ncbi.nlm.nih.gov/protein/225375584?report=genbank&log$=prottop&blast_rank=1&RID=F6PGASAE011) (EC [3.2.1.23](http://www.enzyme-database.org/query.php?ec=3.2.1.23)) and α-galactosidase [ZP_03752801.1](http://www.ncbi.nlm.nih.gov/protein/225375580?report=genbank&log$=prottop&blast_rank=1&RID=F6PKEHZU016) (EC [3.2.1.20](http://www.enzyme-database.org/query.php?ec=3.2.1.20)), respectively, from *Roseburia inulinivorans*. Although, the multi-domain R_01-20 protein could not be expressed in the active form, the mono-domain R_01-21 protein could be produced as monomers of 90 kDa that showed restricted substrate specificity and were only able to hydrolyse *p*NPαGal (**Table S5**). This enzyme was, therefore, determined to be a typical α-galactosidase of the GHF36. The optimal activity was observed within a narrow range of temperatures (40-50ºC) and pH (5.5-6.5) (**Figures S2 and S3**), whereas the activity rapidly decreased at temperatures above 50ºC (60% of the activity was lost at 55ºC).

The insert also contained three sugar transporters (R_01-13, R_01-22 and R_01-27) and two accessory enzymes involved in the conversion of galactose to glucose, namely a galactokinase (R_01-25) and galactose-1-phosphate uridyltransferase (R_01-26), which are most closely related to members of *Clostridium* (59% and 63% identity, respectively). This indicates that the enzyme-producing organism may contain a single cluster of a set of genes encoding proteins for the hydrolysis/synthesis of galactose-containing polymers, which mediate sugar transport and direct galactose monomers to glycolysis in the form of glucose.

*Analysis of the GHs encoded by the sequences in the r_02 fosmid* − The r_02 insert was 26 383 bp in length (G+C content of 50.89%) and contained 25 predicted protein-encoding ORFs. Nine ORFs were unrelated to any known genes, whereas 15 ORFs possessed similarities to identified bacterial genes. Fourteen of the proteins deduced from the latter ORFs revealed high AA sequence identities (74 to 97%) to database entries from *Prevotella ruminicola* (**Table S2B**), a fully sequenced microorganism isolated from the rumen that is thought to facilitate the breakdown of proteins and carbohydrates in this environment. This insert encoded two glycosyl transferases (R_02-11 and R_02-21) and a α-glucanphosphorylase (R_02-20) that are very similar to their homologues from the databases (76-89% AA sequence identity). The tetranucleotide patterns revealed that the r_02 fosmid originates from the chromosome of a *Bacteroidetes* microorganism and is most closely related to the *Prevotella*/*Spirosome* lineage (**Figure S7**).

Two conserved glycosyl hydrolases, one from the GHF51 (EC 3.2.1.55) (R_02-02; 530 AA, a Mm of 59 485 and p*I* of 6.25) and one from the GHF13 (EC 3.2.1.-) (R_02-15; 501 AA, a Mm of 56 950 and p*I* of 5.53), were also found (86 and 79% AA sequence identity to the closest homologues from *P. ruminicola*, respectively). GHF51 is one of the smallest and least diverse families of GHs and includes α-L-arabinofuranosidases (EC [3.2.1.55](http://www.enzyme-database.org/query.php?ec=3.2.1.55)) and endoglucanases (EC [3.2.1.4](http://www.enzyme-database.org/query.php?ec=3.2.1.4)). The activity screens revealed that R_02-02 (monomer of approximately 60 kDa) should be classified as an α-L-arabinopyranosidase because of its ability to hydrolyse *p*NPαAp efficiently. The catalytic efficiency (*k*_cat_/*K*_m_) using *p*NPαAp as the substrate was 3-fold higher than that with *p*NPαAf, with a 26-fold greater *k*_cat_ despite a 12-fold higher *K*_m_ in the former reaction (**Table S5**). The optimum activity for R_02-02 was observed within a narrow range of temperatures (45 and 50ºC) and a narrow pH range (5.0-6.0) (**Figures S2 and S3**). GHF13 includes a large number of -amylases and related enzymes; however, the catalytic properties of R-02-15 could not be confirmed because the corresponding gene could not be functionally expressed in *E. coli*.

*Analysis of the GHs encoded by the sequences in the r_03 fosmid* − The r_03 insert was 19 873 bp in length (G+C content of 62.86%), and 10 putative coding sequences were predicted. The gene arrangement within the cloned fragment is presented in **Figure S1A**. The fragment bears a cluster of carbohydrate metabolism-related genes, including a gene fragment similar to a CBM_51 from a melibiase (R_03-01) that is a putative carbohydrate-binding domain named NPCBM (Pfam_08305) and is found at the N- or C-termini of GHs from different families (**Figure S1A**) [45]. The fragment also contained two consecutive ORFs encoding a GHF43 β-xylosidase (EC [3.2.1.37](http://www.enzyme-database.org/query.php?ec=3.2.1.37)) (R_03-04) and a GHF43 α-L-arabinofuranoside (EC [3.2.1.55](http://www.enzyme-database.org/query.php?ec=3.2.1.55)) (R_03-05). The closest homologues of the two latter proteins were from *Bacteroidetes* (54-58% AA identity) (**Table S2C**) and had deduced molecular masses of 35 572 and 59 988 Da and estimated p*I* values of 5.08 and 7.20, respectively. The cluster also contained accessory enzymes involved in sugar transport (R_03-03) and metabolism, an aldose-1-epimerase (R_03-06), which are most similar to homologous proteins from *Bacteroidetes***.**

Purified recombinant R_03-04 (dimers of 70 kDa) displayed a specificity for *p*NPβX, with a *K*_m_ of 4.12 ± 0.56 mM and a *k*_cat_ of 127.45 ± 11.65 s^-1^ (**Table S5**). Therefore, the enzyme should be classified as a GHF43 β-xylosidase. The activity screens revealed that the R_03-05 enzyme (dimers of 120 KDa) should be classified as a highly efficient GHF43 α-L-arabinofuranoside, as it was able to hydrolyse *p*NPαAf specifically (*K_m_* of 4.9 ± 1.0 mM, *k_cat_* of 708 ± 16 s^-1^) (**Table S5**). Both enzymes showed almost identical pH and temperature profiles, being most active at pH 5.0-7.5 and 45-50ºC.

*Analysis of the GHs encoded by the sequences in the r_05 plasmid* − The insert sequence of r_05 (4638 bp, G+C content of 51.53%) harboured 4 predicted protein-coding ORFs that were significantly similar to database entries from various members of the *Bacteroidetes* (**Figure S1** and **Table S2D**). Among them, two ORFs (R_05-03 and R_05-04) were related to hypothetical proteins with unknown function, whereas R_05-01 belonged to a family of lipoproteins. R_05-02 showed 63% identity to a predicted endoglucanase, CAJ19140.1, from the same library analysed previously [19]. R_05-02 belonged to GHF5 (EC 3.2.1.-), one of the largest and most diverse GH families [46]. This ORF encodes a product of 546 AA, with a deduced molecular mass of 61 422 Da and an estimated p*I* value of 5.31. However, this enzyme could not be expressed in *E. coli* in an active form, and, therefore, its catalytic parameters could not be determined.

*Analysis of the GHs encoded by the sequences in the r_06 plasmid* − The cloned DNA fragment was 5805 bp in length (G+C content of 44.60%) and harboured four ORFs, which are predicted to encode proteins with significant similarities (45-96% AA sequence identity) to deduced proteins from *Prevotella ruminicola* (**Figure S1** and **Table S2E**). R_06-01 encodes a putative 483 AA histidine kinase (R_06-01) with 45% AA identity and 65% AA similarity to homologous proteins. R_06-03 and R_06-04 showed high identities (above 94%) to a D-phosphoglycerate dehydrogenase and a phosphoserine transaminase, respectively. R_06-02 was a putative 690 AA protein with a predicted molecular mass of 78 164 Da and an estimated p*I* value of 4.87, which exhibited a high homology (62% identity and 72% similarity) to a predicted GHF5 endo-β-1,4-xylanase (YP_003575492.1) from *P. ruminicola*. Similar to R_05-02, the activity of this enzyme could not be determined because the enzyme could not be expressed in an active form in *E. coli*.

*Analysis of the GHs encoded by the sequences in the r_07 plasmid* − The insert sequence of r_07 was 4176 bp long, had a molar G+C content of 63.27% and harboured 2 predicted ORFs (**Figure S1** and **Table S2F**). The first ORF encoded a putative 827-AA protein (R_07-01) with a predicted molecular mass of 92 454 Da and an estimated p*I* of 4.92, thus exhibiting a high similarity (42% identity and 59% similarity) to the predicted GHF78 α-L-rhamnosidase, RamA, of *Clostridium stercorarium*. The second predicted ORF encoded a truncated product of 260 AA (R_07-02) with a deduced molecular mass of 29 543 Da and an estimated p*I* value of 8.69. This sequence exhibited a high similarity (42% identity and 56% similarity) to a predicted GHF2 β-galactosidase from the clostridium, *Roseburia intestinales* M50/1.

R_07-01 (monomer of 90 kDa) was shown to catalyse *p*NPαR efficiently, with a *K*_m_ of 3.58 ± 0.70 mM, *k*_cat_ of (2.4 ± 0.2)·10^3^  s^-1^ (**Table S5**) and maximal efficiency between 40-50ºC and within a broad pH range (4.5-9.0) (**Figures S2** and **S3**). Therefore, this enzyme should indeed be classified as an α-L-rhamnosidase of the GHF78 family.

*Analysis of GHs encoded by the sequences in the r_08 plasmid* − The insert sequence of r_08 was 4145 bp in length with a molar G+C content of 63.86% and harboured 2 ORFs (**Figure S1** and **Table S2G**). The first ORF (R_08-01) encodes a putative 922 AA protein with a predicted molecular mass of 103 144 Da and an estimated p*I* value of 5.11. R_08-01 was very similar to GHF78 α-L-rhamnosidases (up to 42% identity and 61% similarity) from various members of *Firmicutes*, such as *Enterococcus casseliflavus* and *Lactobacillus acidophilus*. The second predicted ORF (R_08-02) encodes a 346 AA protein with a predicted molecular mass of 40 359 Da and an estimated p*I* value of 4.95. The second ORF exhibited from 38 to 50% amino acid sequence identity to esterase-lipase and xylanase super-family proteins from *Firmicutes* (*Bacillales*); the highest similarity was with [YP_001698978.1](http://www.ncbi.nlm.nih.gov/protein/169828820?report=genbank&log$=prottop&blast_rank=1&RID=F6YH8CXN014) from *Lysinibacillus sphaericus*. Because enzymes from the esterase family form a part of the enzyme complex acting collectively and synergistically to hydrolyse plant biomass xylans completely to monomers, we characterised this enzyme.

R_08-01 (monomer of approximately 100 kDa) was able to hydrolyse *p*NPαR with a 30-fold higher *k*_cat_ and a similar *K_m_* to those exhibited by the R_07-01 α-L-rhamnosidase, demonstrating in the higher catalytic efficiency of R_08-01 (**Table S5**). Based on its biochemical characteristics and AA homology, the enzyme should be considered a highly efficient GHF78 α-L-rhamnosidase. Its maximal activities were found between 45-50ºC and pH 5.0-6.0 (**Figures S2** and **S3**).

R_08-02 hydrolysed *p*-nitrophenyl propionate (*p*NPP) with the lowest *K_m_,* but it also hydrolysed *p*-nitrophenyl acetate (*p*NPA), methyl ferulate and methyl-*p*-coumarate, with *k*_cat_ relative values of ~8:1:4:4, respectively (**Table S5**). As a consequence, the enzyme was between 3-and 10-fold more catalytically efficient for *p*NPP compared to the other substrates. Neither methyl sinapinate, caffeate nor the longer substrates (*p*NP 5-*O-trans*-feruloyl-α-L-arabinofuranoside and 5-*O*-(*trans*-feruloyl)-α-L-arabinofuranosyl)-(1,3)-β-D-xylopyranosyl-(1,4)-D-xylopyranose) were hydrolysed. According to its substrate specificity, the enzyme was classified as a feruloyl esterase type A. The protein (monomer of 40 kDa) showed maximal activity at 50ºC and was active within a broad pH range from 5.5 to 9.0 (**Figures S2** and **S3**).

**Analysis of the DNA fragments using genome linguistics**

The compositional similarity between the (meta-) genomic fragments and the sequences of related bacterial chromosomes and plasmids was analysed by comparing the frequencies of tetranucleotides in the DNA sequence [47]. From the selected sequences that showed a compositional similarity with the metagenomic fragment, the tetranucleotide patterns revealed that the r_02 fosmid originates from the chromosome of a *Bacteroidetes* microorganism and is most closely related to the *Prevotella*/*Spirosome* lineage (**Figure S7**). However, for the other DNA fragments analysed in the present study, no clear phylogenetic affiliation could be derived using genome linguistics, and the putative phylogenetic affiliation was, thus, inferred from BLASTP bins (**Tables S2** and **S3**).

**3-D modelling analysis of microbial hydrolases from the R library**

We conducted homology-based 3D modelling analyses of the identified proteins; the SWISS-MODEL server was used for building the models**.** The model analysis (**Figure S5**) was complemented with the analysis of the domains (**Figure S4**) present in the full-length polypeptides. The main features of these enzymes are summarised below.

Enzyme R_01-20 clustered within GHF2, but its activity was not experimentally deduced. Our analysis suggested that β-galactosidase from *Bacteroides vulgatus* (PDB: 3gm8; unpublished) would be the best available template (25% sequence identity) to use for the modelling (**Figure S5**). The monomeric unit of the protein is composed by five distinguishable modules, an (α/β)_8_ barrel and four β-sandwich units, as depicted by different colours.

Enzyme R_01-21 is an α-galactosidase belonging to the GHF36 and shares a high sequence identity (41%) with PDB entry 3mi6, an enzyme from *Lactobacillus brevis* with the same function (unpublished). **Figure S5** shows the structure of a monomer of the template protein. From the model and structural analysis, it can be deduced that R_01-21 has a typical GH36 (β/α)_8_ barrel catalytic domain and a second β-jellyroll domain.

Enzyme R_02-02 contains a unique GHF51 domain **and was structurally most similar to an** α-L-arabinofuranosidase (**51% identity)** from *Thermobacillus xylanilyticus* (PDB 2vrq; [48]. The overall structure shows two characteristic domains: a catalytic domain folded into a (β/α)_8_ barrel with one β-sandwich unit and a C-terminal domain that displays jellyroll architecture. Our homology modelling suggested Glu^195^ and Glu^317^ as the putative catalytic residues.

Threading analysis revealed that R_02-15 clearly belongs to GHF13, which includes α-amylases and related enzymes. We used the *Klebsiella* sp. isomaltulose synthase, PDB 1m53 [49], as the best available template, together with 1wzl, 1wza and 1m53 **(18, 19.5 and 16% identity, respectively). From this modelling, we observed that the** R_02-15 enzyme contains a modular structure with a long N-terminal 8-stranded (α/β)_8_ barrel catalytic domain that contains the catalytic core and a C-terminal Greek key β-barrel domain that is interrupted by a ~50 AA calcium-binding domain. Sequence and model analyses identified amino acids Glu^240^ and Asp^211^ as the general nucleophile and the catalytic acid, respectively.

Enzyme R_05-02 (GHF5) was discovered using CMC as the substrate, but its activity could not be demonstrated because it could not be purified in active form. Based on its sequence, we predict that this endoglucanase accepts long-chain substrates, and it shares an identity of 22% with xyloglucanase XG5 from *Paenibacillus pabuli* (PDB 2jep) ]50]. This enzyme displays an open active site groove (with Glu^320^ and Glu^458^ being potential catalytic residues) located upon its (β/α)_8_ fold where xyloglucan may be accommodated.

The R_06-02 protein belongs to the GHF5 family and acts on long polymers, such as CMC, as demonstrated in the initial screening tests; however, its catalytic parameters could not be determined because of our inability to purify it in an active form. Structurally, R_06-02 is highly related to R_05-02, although they share only approximately 8% AA sequence identity. The most similar protein that has been crystallised is the endo-β-1,4-glucanase from *Prevotella bryantii* (PDB 2jep; unpublished). Using PDB 2jep as the template, Glu^179^ and Glu^521^ were found to be the potential catalytic residues.

Enzymes R_07-01 and R_08-01 are α-rhamnosidases belonging to the GHF78 family. An enzyme with the same activity from *Bacteroides thetaiotaomicron* (PDB 3cih, unpublished) was found to be the best (and only) available template. **Figure S5** shows the structure of the template. Not much structural or functional comparative analysis between the metagenomic GHF78 enzymes and 3cih could be performed because the template enzyme sequence has not yet been published. The two conserved motifs typical for GHF78 α-rhamnosidases were identified in these proteins: Pfam Bac_Rhamnosid_N (E value of 8.3e-37-1.6e-41) is located in the N-terminus, and Pfam Bac_Rhamnosid (E value of 6.4e-68-1.7e-154) comprises the C-terminal portion of the polypeptide. The catalytic residues in the active site of R_07-01 (Asp^429^ and Glu^435^) were predicted *in silico* and confirmed experimentally by site-directed mutagenesis.

Analysis of R_08-02 showed that it exhibited the typical canonical α/β-fold that is most **similar (14% identity) to the** acetyl esterase, HerE (PDB 1lzk; [51], which is able to hydrolyse acetyl groups from heroin to yield morphine and from phenyl acetate to yield phenol. This enzyme is encoded by an ORF downstream of that encoding R_08-01 rhamnosidase. Therefore, one could expect these enzymes to be co-expressed and work together in sugar polymer degradation. The primary sequence of this protein shows several conserved domains related to an α/β hydrolase fold and carboxylesterase. One of the conserved Pfam domains indicates that R_08-02 belongs to the dienelactone hydrolase family (Pfam DHL, AA 183-203, E value of 0.024, and AA 277- 328, E value of 2e-05). The active cleft of this enzyme consists of the catalytic residues, Asp^272^, His^304^ and Ser^176^, which constitute the typical catalytic triad of serine esterases, as confirmed by mutation analysis.
