# Supplementary material for: Functional Metagenomics Unveils a Multifunctional Glycosyl Hydrolase from the Family 43 Catalysing the Breakdown of Plant Polymers in the Calf Rumen
Source: PLoS One. 2012 Jun 25;7(6):e38134. doi: 10.1371/journal.pone.0038134 (PMC3382598; doi:10.1371/journal.pone.0038134)
Supplement: References S1 — Complete list of citations for Methods S1 and Text S1. (DOC) [file pone.0038134.s018.doc]

**SUPPORTING REFERENCES**

1. Laemmli UK. (1970). Cleavage of structural proteins during the assembly of the head of bacteriophage T4. Nature 227**:** 680–685.
2. Bradford MM. (1976). A rapid and sensitive method for the quantification of microgram quantities of protein utilizing the principle of protein-dye binding. Anal Biochem 72**:** 248–254.
3. [Rigden DJ](http://www.ncbi.nlm.nih.gov/pubmed?term="Rigden DJ"%5BAuthor%5D). (2005). Analysis of glycoside hydrolase family 98: catalytic machinery, mechanism and a novel putative carbohydrate binding module. [*FEBS Lett*](javascript:AL_get(this, 'jour', 'FEBS Lett.');) **579:** 5466–5472.
4. Elifantz H, Waidner LA, Michelou VK, Cottrell MT, Kirchman DL. (2008). [Diversity and abundance of glycosyl hydrolase family 5 in the North Atlantic Ocean.](http://www.ncbi.nlm.nih.gov/pubmed/18194344) *FEMS Microbiol Ecol* **63:** 316–327.
5. Reva ON, Tümmler B. (2004). Global features of sequences of bacterial chromosomes, plasmids and phages revealed by analysis of oligonucleotide usage patterns. *BMC Bioinformatics* **5:** 90.
6. [Paës G](http://www.ncbi.nlm.nih.gov/pubmed?term="Paës G"%5BAuthor%5D), [Skov LK](http://www.ncbi.nlm.nih.gov/pubmed?term="Skov LK"%5BAuthor%5D), [O'Donohue MJ](http://www.ncbi.nlm.nih.gov/pubmed?term="O'Donohue MJ"%5BAuthor%5D), [Rémond C](http://www.ncbi.nlm.nih.gov/pubmed?term="Rémond C"%5BAuthor%5D), [Kastrup JS](http://www.ncbi.nlm.nih.gov/pubmed?term="Kastrup JS"%5BAuthor%5D), [Gajhede M](http://www.ncbi.nlm.nih.gov/pubmed?term="Gajhede M"%5BAuthor%5D) *et al.* (2008). The structure of the complex between a branched pentasaccharide and *Thermobacillus xylanilyticus* GH-51 arabinofuranosidase reveals xylan-binding determinants and induced fit. [*Biochemistry*](javascript:AL_get(this, 'jour', 'Biochemistry.');) **47:** 7441–7451.
7. Zhang BG, Rouland C, Lattaud C, Lavelle P. (1993). Activity and origin of digestive enzymes in gut of the tropical earthworm *Pontoscolex corethrurus*. *Eur J Soil Biol* **29:** 7–11.
8. [Gloster TM](http://www.ncbi.nlm.nih.gov/pubmed?term="Gloster TM"%5BAuthor%5D), [Ibatullin FM](http://www.ncbi.nlm.nih.gov/pubmed?term="Ibatullin FM"%5BAuthor%5D), [Macauley K](http://www.ncbi.nlm.nih.gov/pubmed?term="Macauley K"%5BAuthor%5D), [Eklöf JM](http://www.ncbi.nlm.nih.gov/pubmed?term="Eklöf JM"%5BAuthor%5D), [Roberts S](http://www.ncbi.nlm.nih.gov/pubmed?term="Roberts S"%5BAuthor%5D), [Turkenburg JP](http://www.ncbi.nlm.nih.gov/pubmed?term="Turkenburg JP"%5BAuthor%5D) *et al*. (2007). Characterization and three-dimensional structures of two distinct bacterial xyloglucanases from families GH5 and GH12. [*J Biol Chem*](javascript:AL_get(this, 'jour', 'J Biol Chem.');) **282:** 19177–19189.
9. Zhu X, Larsen NA, Basran A, Bruce NC, Wilson IA. (2003). [Observation of an arsenic adduct in an acetyl esterase crystal structure.](http://www.ncbi.nlm.nih.gov/pubmed/12421810) *J Biol Chem* **278:** 2008–2014.
